# Intranasal Administration of a Therapeutic HIV Vaccine (Vacc-4x) Induces Dose-Dependent Systemic and Mucosal Immune Responses in a Randomized Controlled Trial

**DOI:** 10.1371/journal.pone.0112556

**Published:** 2014-11-14

**Authors:** Kristin Brekke, Andreas Lind, Carol Holm-Hansen, Inger Lise Haugen, Birger Sørensen, Maja Sommerfelt, Dag Kvale

**Affiliations:** 1 Department of Infectious Diseases, Oslo University Hospital, Oslo, Norway; 2 Norwegian Institute of Public Health, Oslo, Norway; 3 Bionor Pharma, Oslo, Norway; 4 University of Oslo, Oslo, Norway; University of Alabama, United States of America

## Abstract

**Background:**

Vacc-4x, a Gag p24-based therapeutic HIV vaccine, has been shown to reduce viral load set-points after intradermal administration. In this randomized controlled pilot study we investigate intranasal administration of Vacc-4x with Endocine as adjuvant.

**Methods:**

Safety and immunogenicity were tested in patients on effective ART. They were randomized to low, medium or high dose Vacc-4x or adjuvant alone, administered four times at weekly intervals with no booster. Vacc-4x-specific T cell responses were measured *in vitro* by proliferation and *in vivo* by a single DTH skin test at the end of study. Nasal and rectal mucosal secretions were analyzed for Vacc-4x-specific antibodies by ELISA. Immune regulation induced by Vacc-4x was assessed by functional blockade of the regulatory cytokines IL-10 and TGF-β.

**Results:**

Vacc-4x proliferative T cell responses increased only among the vaccinated (p≤0.031). The low dose group showed the greatest increase in Vacc-4x CD8+T cell responses (p = 0.037) and developed larger DTH (p = 0.005) than the adjuvant group. Rectal (distal) Vacc-4x IgA and IgG antibodies also increased (p = 0.043) in this group. In contrast, the high dose generated higher nasal (local) Vacc-4x IgA (p = 0.028) and serum IgG (p = 0.030) antibodies than the adjuvant. Irrespective of dose, increased Vacc-4x CD4+T cell responses were associated with low proliferation (r = −0.82, p<0.001) and high regulation (r = 0.61, p = 0.010) at baseline.

**Conclusion:**

Intranasal administration of Vacc-4x with Endocine was safe and induced dose-dependent vaccine-specific T cell responses and both mucosal and systemic humoral responses. The clinical significance of dose, immune regulation and mucosal immunity warrants further investigation.

**Trial Registration:**

ClinicalTrials.gov NCT01473810

## Introduction

Human Immunodeficiency Virus (HIV) type 1 infection is a challenge for global health [1]. Although many infected individuals have access to well tolerated and effective antiretroviral therapy (ART), alternative treatment strategies are needed, particularly for patients with drug resistance and individuals at high risk of complications related to persistent chronic immune activation [2], [3].

Therapeutic HIV vaccines are designed to induce more effective HIV-specific immune responses [4]. CD4+T cells provide immunological support to HIV-specific CD8+T cells that are crucial for sustained viral control [5]. Effective therapeutic vaccination may delay treatment initiation or supplement ART to further suppress viral replication. Strengthened HIV-specific cellular immune responses attained by therapeutic vaccination may also be essential as part of a future functional cure for HIV [6]–[8]. HIV protein Gag-specific T cell responses are associated with virus control and delayed disease progression [9]. Vacc-4x is a peptide-based therapeutic HIV vaccine corresponding to conserved regions of Gag p24 [10]. Intradermal Vacc-4x with recombinant granulocyte-macrophage colony stimulating factor as adjuvant has been shown to enhance HIV-specific cellular immune responses in patients on ART [11], induce long lasting vaccine-specific T cell memory [12] and lower viral load set-points after interruption of ART [13]. Furthermore, no viral mutations within the conserved HIV p24 sequences were found in vaccinated patients [14].

The primary and secondary objectives of this randomized controlled pilot study were to explore safety and immunogenicity of intranasal administration of Vacc-4x at different doses. Intranasal administration may open for a new and easier means of HIV vaccine delivery, which may be of special interest in resource limited settings. Less trained personnel can administer such vaccines, as has been demonstrated with the oral polio vaccine. In this phase I trial, HIV- infected patients on effective ART were vaccinated by application onto the nasal mucosa with Vacc-4x dissolved in Endocine, an adjuvant developed for mucosal administration [15], [16]. Endocine has previously been shown to be safe and tolerable in humans and the Endocine-adjuvanted whole virus vaccine fulfilled the EMA/CHMP HAI criteria for a seasonal influenza vaccine [17].

Mucosal vaccination may induce both systemic and mucosal immune responses [15], [18], [19]. The latter is relevant for HIV, which is transmitted via mucosa through sexual contact, and later resides and replicates in mucosal tissues. In contrast to other mucosal delivery routes that mostly induce local immune responses, intranasal immunization may generate IgA and IgG antibody responses at distant sites such as cervicovaginal mucosa [20], [21]. Although Vacc-4x was designed to primarily induce systemic cellular immune responses, this study explored whether systemic as well as nasal and rectal humoral responses would be induced following intranasal vaccine delivery.

In chronic HIV infection, HIV-specific T cell responses may be strongly regulated [22], [23], and immune regulatory mechanisms may therefore be important to consider in therapeutic HIV vaccination [24]. As a secondary objective, we therefore assessed vaccine-specific immune regulation mediated by cytokines that potently inhibit Th1 responses as recently described by Lind et al [25]. Our data indicate that both vaccine antigen doses and pre-existing immune regulatory mechanisms to the vaccine antigens may influence the outcome of mucosal therapeutic HIV vaccination.

## Materials and Methods

The protocol for this trial and supporting CONSORT checklist are available as supporting information; see Protocol S1 and Checklist S1.

### Study design and immunization schedule

**Figure 1:**
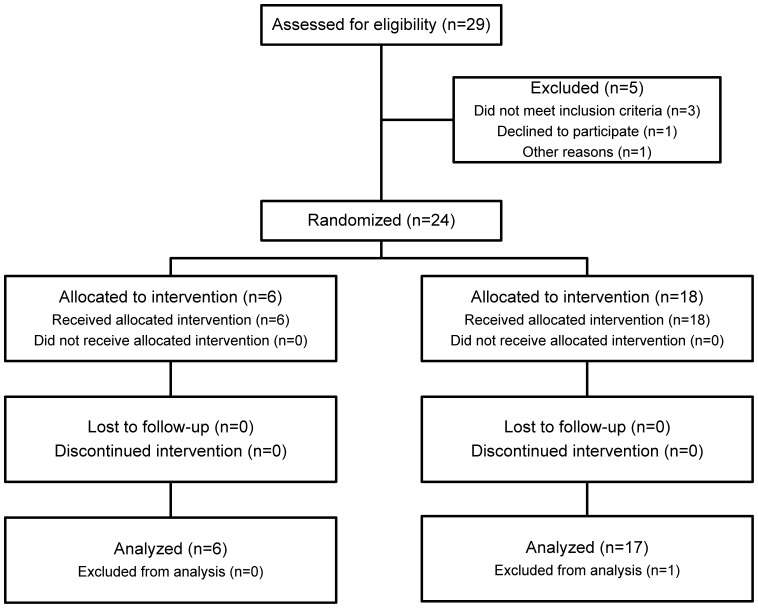
The study CONSORT diagram. Patients were randomized to either Vacc-4x with Endocine as adjuvant (right) or adjuvant alone (left).

This single-blinded, randomized study (Fig. 1) included 24 HIV-infected patients over 18 years of age, infected for more than one year, on effective ART for at least 6 months with viral load <50 copies/ml, CD4+T cell count >400/µl and nadir CD4>200/µl. Exclusion criteria were AIDS-defining illnesses, malignancies, chronic active infections, immunosuppressive therapy, pregnancy, breastfeeding or unacceptable general biochemical and hematological parameters. All patients were selected from the Outpatient Clinic, Department of Infectious Diseases, Oslo University Hospital.

The study was sequential and dose escalating. The random allocation sequence was generated by the monitor. Patients were screened over a three months period and included sequentially according to the allocated dose, interspersed with patients randomized to adjuvant only. Randomization was blinded to patients. The primary endpoint was safety and the number of participants was therefore limited. Regardless, it was possible to statistically analyze secondary endpoints in subgroups. Eighteen patients received Vacc-4x at low (LD, 80 µg Vacc-4x), medium (MD, 400 µg Vacc-4x) or high dose (HD, 1200 µg Vacc-4x) with Endocine as adjuvant, whereas 6 patients received Endocine only. All patients were given 150 µl in each nostril, loaded onto the nasal floor while in an upright position, their head bent slightly backwards to keep the dispersion in the nasal cavity. A total of four doses were administered at weekly intervals with final visit at week 8.

The study was approved by the Norwegian Medicines Agency (EudraCT 2011-000568-80) and the Norwegian Regional Ethics Committee South East (application #2011/2012). Written informed consent was obtained from all participants.

### Peptide design and adjuvant

Vacc-4x is composed of four water-soluble, slightly modified short peptides of 24–27 amino acids, corresponding to conserved regions of the HIV core protein Gag p24 [10].

Endocine is a delivery system specifically developed for intranasal vaccines. It is a lipid-based (monoolein and oleic acid) dispersion with particles less than 100 nm. Endocine was adjusted and tested for solubility and stability alone and in combination with Vacc-4x.

The vaccine was prepared by the Oslo University Hospital pharmacy on the day of administration.

### Monitoring and blood sampling

Adverse events were monitored and clinical examination performed at each visit. Blood samples for safety (general biochemistry and hematology), CD4+ and CD8+T cell counts and HIV RNA were analyzed by conventional methods. Peripheral blood mononuclear cells (PBMC) were obtained from CPT tubes (Becton Dickinson Biosciences, San Jose, CA) (BD). All blood samples were collected prior to immunizations. Serum was processed and stored at -70°C until further analysis.

### Vacc-4x delayed type hypersensitivity test (DTH)

A Vacc-4x DTH was performed at end of study (week 8) by intradermal injection of 400 µg Vacc-4x in 100 µl sterile water as previously described [11]. A positive test was defined as a palpable skin infiltrate with an induration area >10 mm^2^ [11]. Baseline DTH was not performed as intradermal Vacc-4x might interfere with mucosal antigen exposure.

### T cell proliferation and regulation

Fresh PBMC were pulse-labeled with carboxyfluorescein succinimidyl ester (CSFE) (Invitrogen Molecular Probes, OR) at 3 µM for 5 minutes before stimulation with Vacc-4x 15-mer peptide panels at 2.5 µg/ml/peptide as detailed elsewhere [11]. The positive control was 0.5 µg/ml Staphylococcal enterotoxin B (Sigma-Aldrich, Oslo, Norway) (S-A). 250,000 PBMC were cultured in 200 µl serum-free culture medium (Gibco AIM V, Invitrogen/Life technologies, San Diego, CA) at 37°C and 5% CO_2_ in 96-well tissue-culture plates (Nunc, Roskilde, Denmark). 100 µl medium was renewed at day 3, and cells were harvested at day 6 and stained with anti-CD3 Pacific Blue, anti-CD8 AmCyan and 7-aminoactinomycin (7AAD) (BD) to exclude nonviable cells. Vaccine-specific proliferative T cell responses were defined as percentages of live (7AAD-) CFSE^dim^ CD8+ or CD4+ (i.e. CD8-) CD3+T cells. The cut-off for the proliferated CFSE^dim^ T cell subset was set by median fluorescence intensities equal to or below the second proliferated generation. Net responses were calculated by subtracting background proliferation in corresponding unstimulated control cultures.

To quantify antigen-induced cytokine-mediated regulation (R_AC_), inhibitory monoclonal antibodies (mAbs) to interleukin-10 (IL-10) and transforming growth factor-β (TGF-β) at 10 µg/ml final concentration were added to parallel antigen and control cultures according to the manufacturer's instructions (R&D Systems Europe, Abingdon, UK). Regulation was defined as difference in antigen-specific proliferative responses between cultures with or without inhibitory mAbs [26].

Flow cytometry data were obtained with BD FACS Canto II with BD Diva software v6.1.

### Cytokines and chemokines

Cell culture supernatants (100 µl) were harvested 18 hours after stimulation with Vacc-4x peptides and stored at −70°C. Cytokines and chemokines were measured using Bio-Plex XMap technology with Luminex IS 100 (BIO-RAD, CA) and Bio-Plex manager Software v6. StatLIA software package v3 (Brendan Scientific Inc., Carlsbad, CA) was used to calculate sample concentrations.

### Vaccine-specific IgA and IgG antibodies in mucosal secretions and serum

Nasal and rectal mucosal secretions were collected at week 1 and 8. After the administration of approximately 0.4 ml of saline to both cavities using a nasal spray device (Minigrip, Apodan, Copenhagen, Denmark), nasal fluid was absorbed onto dry filter paper collection devices (MucoSafe, Norwegian Institute of Public Health, Oslo, Norway). The filter papers were dried overnight and stored at room temperature (20°C) until extraction [27].

Rectal secretions were collected as described by Kozlowski et al [28]. A sterile swab (Sugi, Kettenbach, Eschenburg, Germany) was pre-moistened with 50 µl saline and placed onto the rectal mucosa 6 cm from the anus for 5 minutes while the patient was lying on one side. The swab was stored at −70°C until extraction.

Nasal and rectal secretions were eluded from filter papers and swabs, respectively, in 1 ml 0.01 M phosphate buffered saline (PBS) with 0.05% Tween 20 using Salivette extraction and centrifuge tubes (Sarstedt AB, Helsingborg, Sweden). Briefly, the roll-shaped swabs provided in the Salivette tubes were removed and discarded. Each filter paper or swab with secretions was placed in a Salivette tube and the cork replaced. PBS was added through the hole in the bottom of the Salivette tube after which the tube was left standing upside down for 15 min to saturate the filter/swab and elude the secretions. The outer centrifuge tube was placed over the inverted Salivette tube and carefully vortexed in this position. Thereafter, the Salivette extraction tube in the outer centrifuge tube was turned right side up and centrifuged at 1000×g for 5 minutes. The extraction tube with the filter paper/swab was removed and discarded, and the extract collected in the centrifuge tube was stored at −20°C until analysis.

IgA and IgG antibodies specific for Vacc-4x and Gag p24 were analyzed by enzyme-linked immunoabsorbent assay (ELISA). Vacc-4x (5 mg/ml) or Gag p24 (1 mg/ml) in sodium carbonate buffer pH 9.5 was used as coating antigen adsorbed on F8 MaxiSorp Immuno Plates (Nunc, Roskilde, Denmark). Non-specific protein-binding sites were blocked with 5% skimmed milk (Oxoid, Basingstoke, UK) in PBS. Two-fold serial dilutions of both mucosal samples and a defined pool of patient secretions in blocking buffer were applied to the plates and incubated overnight at 4°C. The plates were washed 5 times with PBS containing 0.05% Tween-20 and incubated for 1 hour at room temperature with peroxidase-conjugated goat antibodies to human IgA or IgG (S-A). After washing 7 times, bound antibodies were detected with o-phenylene diamine (S-A) in 0.05 M phosphate-citrate buffer, pH 5.0. Optical densities were read at 490 nm in a Thermo max microplate reader (Molecular Devices, Sunnyvale, CA). Standard curves were generated and antibody concentrations in arbitrary units (U) to Vacc-4x or Gag p24 in each sample were determined based on the defined pool of patient secretions [27]. The lowest measurable IgA and IgG antibody levels to these antigens were used as cut-off values. Antibody levels were related to total concentrations of IgA or IgG to adjust for the diluting influence of differences in the amounts of nasal spray used and variations in secretory activity [29]. The adjusted levels were expressed as the ratio of specific IgA or IgG antibodies per weight unit of total IgA or IgG, respectively.

Total IgA and IgG in secretions were determined by ELISA as described [27]. After coating the plates with affinity-purified goat antibodies directed against human IgA (α-chain specific) and IgG (γ-chain specific) (S-A), bound Ig was detected by peroxidase-conjugated goat antibodies to human IgA and IgG (S-A), respectively. Purified human IgA (dimer) and IgG (pooled serum) (Nordic Immunological Laboratories, Tilburg, the Netherlands) were used as standards.

Serum samples were analyzed by ELISA for Vacc-4x and Gag p24 IgG antibodies based on a defined pool of patient sera.

### Statistical methods

Statistical analysis and graphics were performed by Statistica v7 (StatSoft, Tulsa, OK) using non-parametrical statistics. Mann-Whitney U test was applied for group-wise comparisons, Wilcoxon matched pairs test for dependent variables, and Spearman rank for correlations. Fisher exact test was used to test proportional differences. All data are presented as medians (interquartile ranges, IQR). p-values <0.05 were considered significant.

## Results

### Safety and tolerability

A total of 23 patients (Table 1) completed the study. One patient in the HD group was hospitalized due to aggravation of mild pre-study myositis and taken off ART with improved condition before the final visit. As a result of the ART interruption the patient was excluded from the data analysis. No serious adverse event related to study vaccine was reported. Mild and transient (<1 hour) soreness at the application site was frequently reported in all groups. Mild flu-like symptoms, upper respiratory infections and viral gastroenteritis were occasionally noted but were not related to the vaccine. Overall, the vaccine was well tolerated with no major changes in vital signs, laboratory parameters, HIV RNA, CD4+ or CD8+T cell counts.

**Table 1:** Cohort characteristics.

	Adjuvant (n = 6)	Vaccine (n = 17)	p
Male: Female	6∶0	16∶1	
Caucasian: Black	6∶0	16∶1	
Age (years)	43.7 (39.7–56.4)*	48.8 (39.5–4.4)	ns
Time since diagnosis (years)	8.9 (4.0–10.9)	7.5 (5.1–12.4)	ns
Time on ART (years)	3.1 (2.6–4.6)	3.5 (2.4–9.4)	ns
Nadir CD4 (cells/µl)	254 (200–280)	301 (240–362)	ns
CD4 (cells/µl)	677 (578–748)	775 (660–985)	ns
CD8 (cells/µl)	1104 (900–1374)	909 (778–1123)	ns
HIV RNA (copies/ml)	<20	<20	ns

^*^Median (interquartile range).

### Dose-dependent Vacc-4x DTH induration at end of study

**Figure 2:**
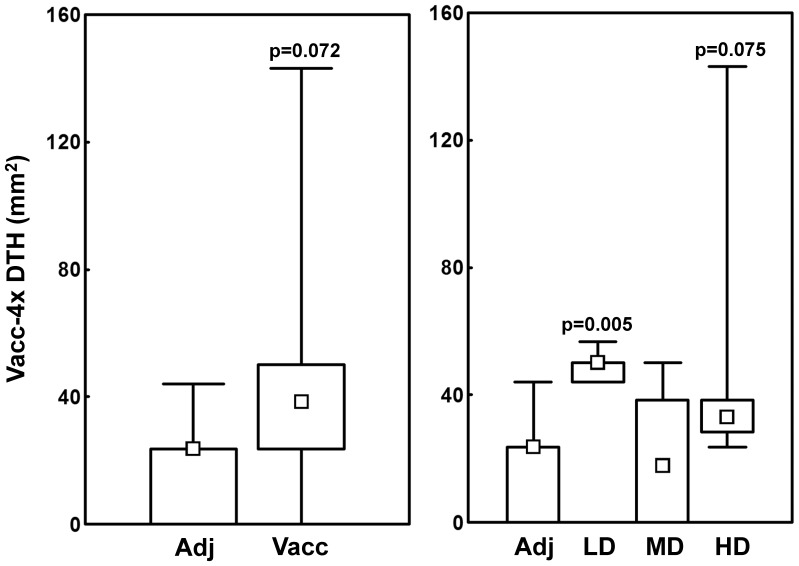
Vacc-4x DTH skin tests at week 8. Induration areas in the adjuvant and the vaccinated group (left panel) and the four dose groups (right panel). Adj  =  adjuvant, LD  =  low, MD  =  median and HD  =  high dose. Data are given as medians, interquartile and overall ranges. Differences between dose groups and the adjuvant group with p-values less than 0.10 are indicated (Mann-Whitney U test).

At end of study, four weeks after the last vaccine or adjuvant dose, a Vacc-4x DTH skin test was performed to assess cellular immune responses *in vivo*. Fifteen (88%) of the 17 remaining subjects in the vaccinated group had a positive DTH compared to 4 (67%) in the adjuvant group (p = 0.271). DTH indurations tended to be larger for the Vacc-4x group (median 39 mm^2^) compared to the adjuvant group (median 24 mm^2^; p = 0.072). Dose-related differences were observed; the LD group presented significantly larger infiltrates (median 50 mm^2^) than the adjuvant group (p = 0.005) with a similar trend for the HD group (median 33 mm^2^; p = 0.075) (Fig. 2).

### Vacc-4x-induced changes in vaccine-specific CD8+ and CD4+T cell responses

**Figure 3:**
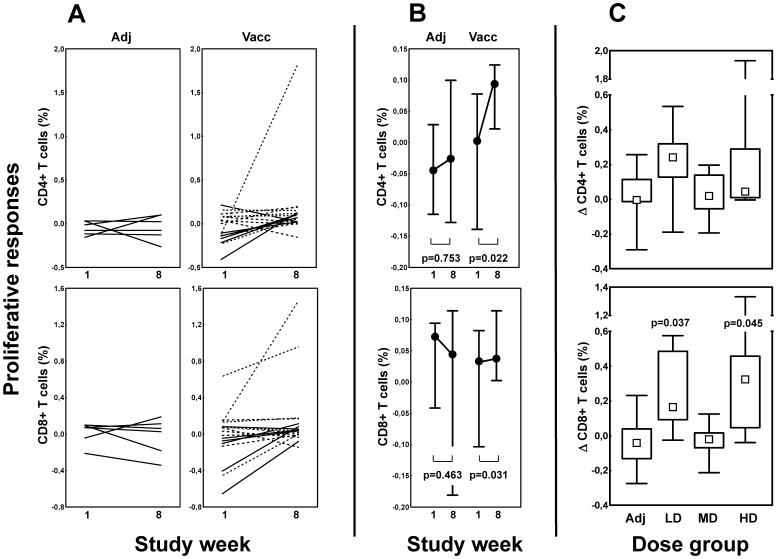
Vacc-4x proliferative T cell responses. **A.** Individual responses at baseline (week1) and end of study (week 8) for CD4+ (upper panels) and CD8+ (lower panels) T cell subsets in the adjuvant (left) and the vaccinated (right) group. Doses are indicated as solid (low), dashed (median) or dotted (high dose) lines, respectively. **B.** Responses in both T cell subsets at baseline (week 1) and end of study (week 8) in the adjuvant (Adj) and the vaccinated group (Vacc), respectively. Changes within each group are indicated (Wilcoxon matched pairs test). **C.** Changes (Δ) in responses from baseline to end of study in the four dose groups. Adj  =  adjuvant, LD  =  low, MD  =  median and HD  =  high dose. Data are given as medians, interquartile and overall ranges. Significant differences between dose groups and the adjuvant group are indicated (Mann-Whitney U test).

At baseline, no significant differences in Vacc-4x-specific proliferative T cell responses were observed between any of the four groups. The individual responses over time are shown in Fig. 3A; increased responses were seen in 12 (71%) of the 17 vaccinated patients. Proliferative T cell responses improved in both T cell subsets (p≤0.031, Wilcoxon matched pairs test) from baseline to week 8 (Fig. 3B). Proliferative CD8+T cell responses increased in both the LD and HD groups compared to the adjuvant group (p = 0.037 and 0.045, respectively) (Fig. 3C).

A selection of soluble mediators was measured in cell culture supernatants after stimulation for 18 hours to assess early T cell activation. At baseline, proliferative T cell responses correlated with levels of the proinflammatory chemokine MIP-1β (CD4+r = 0.47, p = 0.024) and inversely with the Th2 cytokine IL-13 (CD8+r = −0.52, p = 0.009). At end of study, supernatant levels of the Th1 cytokine TNF-α were higher than at baseline in the vaccinated (p = 0.027) but not in the adjuvant group (p = 0.463). Moreover, Vacc-4x-specific proliferative T cell responses correlated with the concentration of RANTES in the vaccinated (CD4+r = 0.72, p = 0.001; CD8+r = 0.49, p = 0.048) but not in the adjuvant group (CD4+/CD8+r = 0.14, p = 0.787) (data not shown).

### Improved Vacc-4x T cell responses predicted by low T cell proliferation and high T cell regulation at baseline

Irrespective of dose, increased Vacc-4x responses were most pronounced in subjects with low baseline responses. This was indicated by an inverse correlation between baseline proliferation and change in proliferative T cell responses induced by Vacc-4x, particularly in the CD4+T cell subset (r = -0.82, p<0.001).

**Figure 4:**
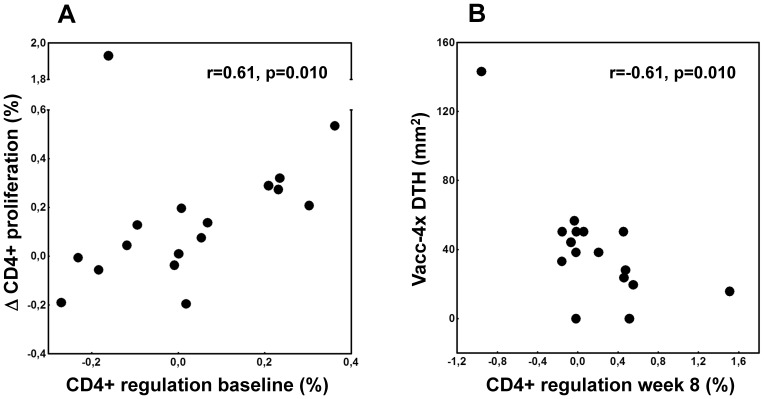
Relation between regulation and *in vitro* and *in vivo* T cell responses. **A.** Relation between Vacc-4x-induced regulation at baseline and change (Δ) in Vacc-4x proliferative T cell responses from baseline to end of study in the CD4+T cell subset (vaccinated group). Spearman rank correlation and p-value are indicated. **B.** Relation between Vacc-4x-induced regulation in the CD4+T cell subset and Vacc-4x DTH induration at week 8 (vaccinated group). Spearman rank correlation and p-value are indicated.

Vacc-4x proliferative responses equal to or even lower than the background proliferation in corresponding unstimulated control cultures might be related to immune regulation. Therefore, Vacc-4x-induced regulation (R_AC_) was explored. More subjects with negative baseline responses relative to control cultures had detectable Vacc-4x-induced regulation than subjects with positive baseline responses [CD4+; 83% (n = 12) vs 27% (n = 11), p = 0.012 and CD8+; 80% (n = 10) vs 15% (n = 13), p = 0.003]. Moreover, Vacc-4x-induced regulation at baseline correlated with the change in Vacc-4x-specific proliferation in the CD4+T cell subset (r = 0.61, p = 0.010) (Fig. 4A), suggesting that regulation at baseline was beneficial. In group-wise analysis this association was significant only for the LD group (r = 0.83, p = 0.042).

We also examined factors related to change in Vacc-4x regulation over time in response to vaccination. Increased regulation in the CD8+T cell subset was associated with high baseline Vacc-4x-specific proliferation (r = 0.74, p<0.001) and low baseline regulation (r = −0.71, p = 0.001). Dose-dependent regulation seemed to occur as an increase in R_AC_ was found only in the HD (p = 0.043) and possibly MD (p = 0.075) groups, but not in the LD or adjuvant groups (data not shown).

Finally, when the Vacc-4x DTH *in vivo* responses, the proliferative responses and regulation *in vitro* were compared, the DTH responses were best associated with Vacc-4x-induced regulation in the CD4+T cell subset (r = −0.61, p = 0.010) (Fig. 4B).

### Dose-dependent Vacc-4x humoral responses in mucosal secretions and serum

Nasal and rectal mucosal secretions were collected at baseline and end of study from 23 (100%) and 22 (96%) of the participants, respectively. IgA and IgG antibodies to Vacc-4x and HIV p24 protein were detected in all patient sera at baseline, but not in HIV-seronegative control samples (data not shown).

**Figure 5:**
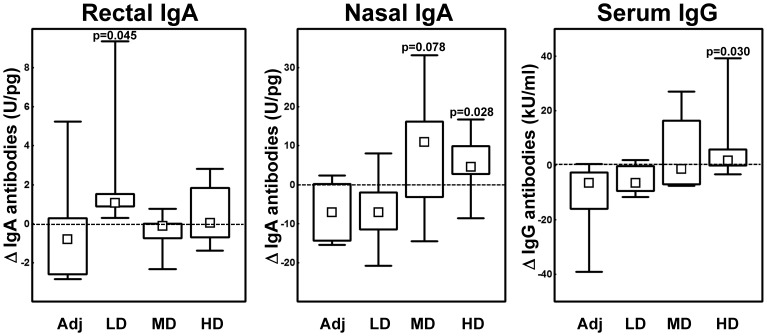
Vacc-4x Ig antibody levels. Changes (Δ) from baseline to end of study in rectal IgA, nasal IgA and serum IgG antibody levels in the four dose groups. Adj  =  adjuvant, LD  =  low, MD  =  median and HD  =  high dose. IgA antibody levels are adjusted to total IgA in rectal and nasal samples (please note different scales). Data are given as medians, interquartile and overall ranges. Differences between dose groups and the adjuvant group with p-values less than 0.10 are indicated (Mann-Whitney U test). Kruskal-Wallis ANOVA analysis for all four groups yielded p = 0.044 (rectal IgA), p = 0.093 (nasal IgA) and p = 0.067 (serum IgG).

In rectal mucosal samples at baseline, Vacc-4x IgA and IgG antibodies were detected in 91% and 64% (i.e. above assay cut-off), respectively, and correlated with the anti-p24 levels (IgA r = 0.53, p = 0.012; IgG r = 0.78, p<0.001). Nasal Vacc-4x IgA and IgG antibody levels were above cut-off in 78% and 61%, respectively. During the study period, a significant increase in distal (rectal) mucosal Vacc-4x IgA and IgG antibodies was seen only among LD patients (both p = 0.043, Wilcoxon matched pairs test). Moreover, the LD group was the only one where rectal Vacc-4x IgA antibodies increased significantly compared to the adjuvant group (Fig. 5, Mann-Whitney U test). In contrast, an increase in local (nasal) IgA antibodies was seen in the two higher dose groups compared to the adjuvant group (Fig. 5) and the baseline levels as well as changes from baseline were larger than in rectal secretions. In serum, Vacc-4x IgG antibody levels increased significantly only in the HD group compared to the adjuvant group (p = 0.030) (Fig. 5).

Finally, changes in cellular and humoral responses to Vacc-4x after vaccination were compared. Negative correlations were found between systemic proliferative T cell responses and nasal Ig antibody levels (IgG r≤−0.48, p≤0.05, CD4+ and CD8+; IgA r = −0.47, p = 0.058, CD8+) as well as serum Vacc-4x IgA responses (r≤−0.58, p≤0.015) (data not shown).

## Discussion

Mucosal therapeutic vaccination has been poorly explored in humans. Leroux-Roels et al. [30] recently described parenteral prime and mucosal boosting with Env gp41 virosomes for prophylactic use in healthy women based on promising data from non-human primates [31]. In this study intranasal administration of Vacc-4x with Endocine as a local adjuvant was well tolerated. Without a booster phase, Vacc-4x-specific proliferative T cell responses for both T cell subsets increased among the vaccinated group from baseline to end of study as compared to the control group that only received adjuvant.

Despite the limited number of patients receiving each dose, dose-dependent differences in both cellular and humoral vaccine-specific responses were observed. Vacc-4x CD8+T cell proliferative responses increased among low dose patients from baseline to end of study and Vacc-4x DTH infiltrates were larger after vaccination than among patients in the adjuvant group. Furthermore, the low dose induced better distal (rectal) Vacc-4x humoral responses, although at lower levels than in nasal secretions. In contrast, the high dose generated higher local (nasal) IgA and serum IgG levels. These observations suggest that the mucosal HIV antigen dose may be one factor directing the immune response modality in chronically infected patients.

Inducing functional HIV-specific cellular immune responses at mucosal sites might assist in controlling viral replication [32]. From a therapeutic HIV vaccine perspective, targeting chronic HIV infection in the mucosa may be important [32]. The role of mucosal antibodies is less clear. Mucosal HIV-specific IgA has proven protective in individuals who have repeatedly been exposed to HIV [33], [34] but these responses are scarce in chronically infected patients [35]. Since Vacc-4x reduces viral load set points after treatment interruption [13], future vaccine studies should provide an opportunity to study mucosal biopsies and viral load measured by an ultra-sensitive HIV RNA assay.

In chronic infection, HIV-specific T cell responses are often dysfunctional [23], [36] and hampered by early clonal T cell loss [37]. Therefore, many chronically infected patients have insufficient *in vitro* T cell responses to Gag. Results from our present study also showed weak responses to the Vacc-4x Gag p24 consensus peptides. The T cell responses induced by intranasal administration of Vacc-4x were generally weaker than in former trials, but patients in the former studies were given more immunizations as well as boosters. Additional booster immunizations may have contributed to the durable T cell responses observed [11], [12]. Regardless, the relevance of vaccine-induced T cell responses *in vitro* was supported by *in vivo* data that showed larger Vacc-4x DTH infiltrates among the vaccinated as compared to the adjuvant group. The Vacc-4x DTH skin test is a reliable indicator of cellular immune responses *in vivo* [38].

T cell proliferation was the main *in vitro* outcome in accordance with former Vacc-4x trials. Proliferation is fundamental for effective immune responses and is a standard and robust method for characterizing T cell functionality [39]–[42]. We have presented proliferation as raw data that resulted in some cases of negative proliferative responses, i.e. negative net responses were obtained when adjusting for background proliferation in unstimulated control cultures. There are several possible explanations for these negative values. First, toxic byproducts from the peptide synthesis process could be present, but this is unlikely because even positive responses were obtained with the same peptide batches. Second, assay variation around zero may occur among patients with low vaccine-specific responses. However, negative responses were repeatedly found in the same patients. Alternatively, Vacc-4x T cell responses in some patients are strongly regulated by mechanisms that reduce even the background proliferation in Vacc-4x-stimulated cultures, resulting in negative net proliferation relative to the corresponding unstimulated control culture. This is in accordance with variable frequencies and suppressive functions of specific regulatory T cells after therapeutic vaccination with autologous dendritic cells loaded with HIV peptides [24]. Based on recent observations [25], [26], we therefore tested immune regulation by blocking IL-10, an anti-inflammatory cytokine, and TGF-β, a regulatory protein, in Vacc-4x-stimulated cell cultures. This regulatory parameter has been associated with rapid disease progression in untreated individuals [26] and might explain the inconsistent effects observed following two Vacc-4x booster doses in patients on ART [25]. In the present study we found that Vacc-4x-induced regulation could explain most cases of negative proliferation.

Vacc-4x regulation was predictive in other respects as well. Vacc-4x DTH infiltrates were inversely correlated with concurrent regulation after vaccination. Moreover, patients who showed the greatest improvement in proliferative T cell responses had higher levels of regulation at baseline. This may indicate that the balance between Vacc-4x-induced T cell activation and regulation may be altered by intranasal administration of Vacc-4x. Given that our exploratory regulatory parameter is clinically relevant, patients with vaccine-induced regulation at baseline might profit from intranasal therapeutic vaccination. In contrast, we observed cases of enhanced regulation after late boosters in intradermal immunization [25]. Altogether, our observations indicate that it might be advantageous to select candidates for therapeutic vaccination trials based on both specific T cell responses and regulation at screening. Furthermore, these parameters might be valuable in monitoring vaccine responses and thereby individualize therapeutic vaccination.

The choice of Vacc-4x doses given was based in part on experience from previous Vacc-4x trials with intradermal injections even though the low dose in this study was only 1/5 of the intradermal low dose [11]. The finding that low dose generated the best proliferative T cell responses but no increase in local IgA antibody levels may indicate that this dose induced a more Th1-like response than the higher doses of Vacc-4x. The potential for different antigen doses to direct immune responses in nasal therapeutic vaccination remains to be determined.

In conclusion, we showed that intranasal administration of Vacc-4x with Endocine as adjuvant was safe and induced dose-dependent vaccine-specific T cell responses and both mucosal and systemic humoral responses. The clinical significance of these findings should be addressed in larger cohorts. The impact of vaccine antigen doses and immune regulatory mechanisms warrants further investigation.

## Supporting Information

Checklist S1 **CONSORT checklist.**
(DOC)

Protocol S1 **Clinical trial protocol.**
(DOC)
