# Supplementary material for: Intranasal Administration of a Therapeutic HIV Vaccine (Vacc-4x) Induces Dose-Dependent Systemic and Mucosal Immune Responses in a Randomized Controlled Trial
Source: PLoS One. 2014 Nov 14;9(11):e112556. doi: 10.1371/journal.pone.0112556 (PMC4232368; doi:10.1371/journal.pone.0112556)
Supplement: Protocol S1 — Clinical trial protocol. (DOC) [file pone.0112556.s002.doc]

Clinical Research Protocol

| Protocol Title: | IMMUNOTHERAPY OF HIV-INFECTED PATIENTS  A single-blinded, randomized, immunogenicity, pilot study of intranasal administration of Vacc-4x with Endocine as adjuvant. |
| --- | --- |
| Protocol number: | CTN-Vacc-4x/L3-2011/1 |
| Version: | 3 |
| Drug products: | Vacc-4x |
| Drug substance: | Vacc-4x: A formulation of four synthetic peptides (Vacc-10, Vacc-11, Vacc-12, and Vacc-13)  Endocine adjuvant: a lipid based nano-emulsion |
| EudraCT Number | 2011-000568-80 |
|  |  |
| Protocol date: | 2012-05-20 |

CONFIDENTIAL

# Protocol Signature Page

| Protocol Title: | IMMUNOTHERAPY OF HIV-INFECTED PATIENTS  A single-blinded, randomized, immunogenicity, pilot study of intranasal administration of Vacc-4x with Endocine as adjuvant. | |
| --- | --- | --- |
| Protocol Number: CTN-Vacc-4x/L3-2011/1 | |  |

**Sponsor and Principal Investigator (hereafter Principal Investigator (PI))**

Name: Dag Kvale MD, Professor

Address: Oslo University Hospital

NO-0450 Oslo, Norway

Phone: +47 22 11 91 53

E-mail: dag.kvale@medisin.uio.no

Sign: _______________________________ Date:_________________________

**Investigational Drug Substance Suppliers**

Vacc-4x: Bionor Pharma ASA, Kronprinsesse Märthas Plass 1, P.O.Box 1477 Vika, NO-0116, Norway

Eurocine adjuvant: Eurocine Vaccines AB, Karolinska Institutet Science Park, Fogdevreten 2, 171 65 Solna, Sweden

Table of Contents

Page

1 Protocol Signature Page [2](#__RefHeading___Toc289425492)

2 introduction [10](#__RefHeading___Toc289425493)

2.1 Background information [10](#__RefHeading___Toc289425494)

2.2 Investigational product [11](#__RefHeading___Toc289425495)

2.3 Nonclinical studies [12](#__RefHeading___Toc289425496)

2.4 Clinical Studies [12](#__RefHeading___Toc289425497)

2.5 Adjuvant [12](#__RefHeading___Toc289425498)

2.6 Rationale for the Study [13](#__RefHeading___Toc289425499)

2.6.1 Dose [13](#__RefHeading___Toc289425500)

2.6.2 Immunization schedule [14](#__RefHeading___Toc289425501)

2.6.3 Use of adjuvant Endocine [14](#__RefHeading___Toc289425502)

2.7 Potential Risks and Benefits [14](#__RefHeading___Toc289425503)

3 Study objectives AND ENDPOINTS [14](#__RefHeading___Toc289425504)

3.1 Study objectives [14](#__RefHeading___Toc289425505)

3.1.1 Primary objective [14](#__RefHeading___Toc289425506)

3.1.2 Secondary objectives [14](#__RefHeading___Toc289425507)

3.1.3 Exploratory objectives [15](#__RefHeading___Toc289425508)

3.2 Study endpoints [15](#__RefHeading___Toc289425509)

3.2.1 Safety endpoints [15](#__RefHeading___Toc289425510)

3.2.2 Efficacy endpoints [15](#__RefHeading___Toc289425511)

3.2.3 Exploratory endpoints [15](#__RefHeading___Toc289425512)

4 study design [16](#__RefHeading___Toc289425513)

4.1 Overall study design and flow chart [16](#__RefHeading___Toc289425514)

4.2 Rationale for study design and treatment regimens [17](#__RefHeading___Toc289425515)

5 Selection and withdrawal of subjects [18](#__RefHeading___Toc289425516)

5.1 Subject screening, enrollment, and identification log [18](#__RefHeading___Toc289425517)

5.2 Inclusion criteria [18](#__RefHeading___Toc289425518)

5.3 Exclusion criteria [18](#__RefHeading___Toc289425519)

5.4 Withdrawal of subjects [19](#__RefHeading___Toc289425520)

5.4.1 Vaccine toxicity management [19](#__RefHeading___Toc289425521)

6 Treatment of Subjects [20](#__RefHeading___Toc289425522)

6.1 Study drug and dosages [20](#__RefHeading___Toc289425523)

6.1.1 Study drug description [20](#__RefHeading___Toc289425524)

6.1.2 Dosage and administration of study drug [21](#__RefHeading___Toc289425525)

6.1.3 Rationale for dosing regimen [22](#__RefHeading___Toc289425526)

6.1.4 Subject compliance [22](#__RefHeading___Toc289425527)

6.1.5 Overdose and toxicity management [22](#__RefHeading___Toc289425528)

6.2 Concurrent medications and Non‑drug therapies [22](#__RefHeading___Toc289425529)

6.2.1 Permitted medications [22](#__RefHeading___Toc289425530)

6.2.2 Prohibited medications [22](#__RefHeading___Toc289425531)

6.3 Study drug management [23](#__RefHeading___Toc289425532)

6.3.1 Packaging of study drug [23](#__RefHeading___Toc289425533)

6.3.2 Labeling of study drug [23](#__RefHeading___Toc289425534)

6.3.3 Preparation of study drug [23](#__RefHeading___Toc289425535)

6.3.4 Preparation of DTH-test [23](#__RefHeading___Toc289425536)

6.3.5 Storage of study drug [24](#__RefHeading___Toc289425537)

6.3.6 Study drug shipping and handling [24](#__RefHeading___Toc289425538)

6.3.7 Study drug accountability [24](#__RefHeading___Toc289425539)

6.4 Procedures for assigning subjects to treatment groups [25](#__RefHeading___Toc289425540)

7 Study Procedures [25](#__RefHeading___Toc289425541)

7.1 Time and events schedule [25](#__RefHeading___Toc289425542)

7.2 Subject informed consent [25](#__RefHeading___Toc289425543)

7.3 Procedures by main study phases [26](#__RefHeading___Toc289425544)

7.3.1 Pre-treatment period [26](#__RefHeading___Toc289425545)

7.3.2 Treatment Period [26](#__RefHeading___Toc289425546)

7.3.3 Follow‑up period [28](#__RefHeading___Toc289425547)

7.4 Early withdrawal visit [29](#__RefHeading___Toc289425548)

7.5 Subject discontinuation (lost to follow‑up) [29](#__RefHeading___Toc289425549)

8 Study Assessments and Measurements [29](#__RefHeading___Toc289425550)

8.1 Demographic and screening assessments [29](#__RefHeading___Toc289425551)

8.2 Efficacy assessments [30](#__RefHeading___Toc289425552)

8.2.1 Delayed type hypersensitivity test administration [30](#__RefHeading___Toc289425553)

8.2.2 CD4 counts [30](#__RefHeading___Toc289425554)

8.2.3 T-cell response [30](#__RefHeading___Toc289425555)

8.2.4 Exploratory analyses [30](#__RefHeading___Toc289425556)

8.3 Safety assessments [31](#__RefHeading___Toc289425557)

8.3.1 Medical history [31](#__RefHeading___Toc289425558)

8.3.2 Physical examination [31](#__RefHeading___Toc289425559)

8.3.3 Vital signs [31](#__RefHeading___Toc289425560)

8.3.4 Clinical laboratory tests [31](#__RefHeading___Toc289425561)

8.3.5 HIV viral load (HIV-1 RNA) [32](#__RefHeading___Toc289425562)

9 Adverse Event Management [32](#__RefHeading___Toc289425563)

9.1 Non‑serious and serious adverse events [32](#__RefHeading___Toc289425564)

9.1.1 Definition of an adverse event [32](#__RefHeading___Toc289425565)

9.1.2 Definition of a serious adverse event [33](#__RefHeading___Toc289425566)

9.1.3 Clinical laboratory abnormalities and other abnormal assessments [34](#__RefHeading___Toc289425567)

9.1.4 Recording of adverse events and serious adverse events [34](#__RefHeading___Toc289425568)

9.1.5 Follow-up of adverse events and serious adverse events [36](#__RefHeading___Toc289425569)

9.1.6 Reporting of all serious adverse events and any adverse events resulting in study discontinuation [36](#__RefHeading___Toc289425570)

9.1.7 Post-study adverse events or serious adverse events [37](#__RefHeading___Toc289425571)

9.2 Overdose [37](#__RefHeading___Toc289425572)

10 data collectionData Collection [37](#__RefHeading___Toc289425573)

10.1 Data collection [37](#__RefHeading___Toc289425574)

10.2 Correcting the case report forms [38](#__RefHeading___Toc289425575)

10.3 Data processing [38](#__RefHeading___Toc289425576)

11 sTATISTICal methods and planned analyses [38](#__RefHeading___Toc289425577)

11.1 Determination of sample size [38](#__RefHeading___Toc289425578)

11.2 Randomization [38](#__RefHeading___Toc289425579)

11.3 Populations to be analyzed [39](#__RefHeading___Toc289425580)

11.4 Handling of missing data [39](#__RefHeading___Toc289425581)

11.5 Subject accountability [39](#__RefHeading___Toc289425582)

11.5.1 Treatment compliance [39](#__RefHeading___Toc289425583)

11.6 Statistical methods [39](#__RefHeading___Toc289425584)

11.6.1 Analysis of demographic and subject characteristics [39](#__RefHeading___Toc289425585)

11.6.2 Safety analysis [40](#__RefHeading___Toc289425586)

11.6.3 Efficacy analysis [41](#__RefHeading___Toc289425587)

12 study management and ethical and regulatory requirements [41](#__RefHeading___Toc289425588)

12.1 Regulatory approval and Good Clinical Practice [41](#__RefHeading___Toc289425589)

12.2 Deviations from the protocol and protocol amendments [41](#__RefHeading___Toc289425590)

12.3 Discontinuation of study [42](#__RefHeading___Toc289425591)

12.4 End of study [42](#__RefHeading___Toc289425592)

12.5 Study records retention and direct access to source documents [42](#__RefHeading___Toc289425593)

12.6 Investigator responsibilities [43](#__RefHeading___Toc289425594)

12.6.1 Subject information and informed consent [43](#__RefHeading___Toc289425595)

12.6.2 Independent ethics committee approval and other institutional requirements [44](#__RefHeading___Toc289425596)

12.6.3 Curriculum vitae [44](#__RefHeading___Toc289425597)

12.6.4 Laboratory certification and normal values [44](#__RefHeading___Toc289425598)

12.6.5 Delegation of investigator responsibilities [44](#__RefHeading___Toc289425599)

12.6.6 Liability and insurance [44](#__RefHeading___Toc289425600)

12.7 Study monitoring and auditing [45](#__RefHeading___Toc289425601)

12.8 Quality assurance [45](#__RefHeading___Toc289425602)

12.9 Study termination and site closure [46](#__RefHeading___Toc289425603)

12.10 Site termination [46](#__RefHeading___Toc289425604)

13 disclosure of data [47](#__RefHeading___Toc289425605)

13.1 Confidentiality [47](#__RefHeading___Toc289425606)

13.2 Publication [47](#__RefHeading___Toc289425607)

14 Investigator’s protocol agreement [48](#__RefHeading___Toc289425608)

15 References [49](#__RefHeading___Toc289425609)

16 appendices [51](#__RefHeading___Toc289425610)

16.1 Division of AIDS Table for Grading Severity of Adult and Pediatric Adverse Events, December 2004 [51](#__RefHeading___Toc289425611)

16.2 AIDS-Defining Illnesses [51](#__RefHeading___Toc289425612)

List of Abbreviations

| **Abbreviation** | Definition/Term |
| --- | --- |
| AE | Adverse Event |
| AIDS | Acquired Immunodeficiency Syndrome |
| ALT | Alanine Transaminase (SGPT) |
| ALP | Alkaline Phosphatase |
| ART | Antiretroviral Therapy |
| AST | Aspartate Transaminase (SGOT) |
| CRF | Case Report Form |
| CRP | C-reactive Protein |
| DTH | Delayed type Hypersensitivity |
| EDTA | Ethylenediamine Tetra acetic Acid |
| ELISPOT | Enzyme-linked Immunosorbent Spot Assay |
| EU | European Union |
| Eur Pharm | European Pharmacopoeia |
| γ-GT | Gamma Glutamyl Transferase |
| GCP | Good Clinical Practice |
| GM-CSF | Granulocyte Macrophage Colony Stimulating Factor |
| GMP | Good Manufacturing Practice |
| HIV | Human Immunodeficiency Virus |
| HLA | Human Leukocyte Antigen |
| ICF | Informed Consent Form |
| ICH | International Conference on Harmonization |
| IEC | Independent Ethics Committee |
| IMP | Investigational Medicinal Product |
| IND | Investigational New Drug |
| LD | Lactate Dehydrogenase |
| MedDRA | Medical Dictionary for Regulatory Activities Terminology |
| PBL | Peripheral Blood Lymphocyte |
| PI | Principal Investigator / Sponsor |
| SAE | Serious Adverse Event |
| SOC | System Organ Class |
| Th1 | T-helper Cell Type 1 |
| Th2 | T-helper Cell Type 2 |
| ULN | Upper Limit of Normal |
| USP | United States Pharmacopoeia |
| WBC | White Blood Cells |
| WFI | Water For Injection |
| WHO | World Health Organization |
|  |  |
|  |  |

Protocol Synopsis

| Protocol Title: | | IMMUNOTHERAPY OF HIV-INFECTED PATIENTS  A single-blinded, randomized, immunogenicity, pilot study of intranasal administration of Vacc-4x with Endocine as adjuvant. | | |
| --- | --- | --- | --- | --- |
| Protocol Number: | | CT -BI-Vacc-4x/L3-2011/1 | | |
| **Name of Sponsor & Investigatio­nal Drug Substance Suppliers:** | Sponsor/Investigator: Prof. Dag Kvale, Oslo University Hospital  Vacc-4x: Bionor Pharma ASA, Kronprinsesse Märthas Plass 1, P.O.Box 1477 Vika, NO-0116, Norway  Eurocine adjuvant: Eurocine Vaccines AB, Karolinska Institutet Science Park, Fogdevreten 2, 171 65 Solna, Sweden | | | |
| Drug Substance: | Vacc-4x: A formulation of four synthetic peptides (Vacc-10, Vacc-11, Vacc-12, and Vacc-13).  Endocine adjuvant: A lipid based nano-emulsion consisting of mono-olein and oleic acid in 0.15 M NaOH at pH 8.0. | | | |
| Investigational Drug Product: | Vacc-4x with Endocine adjuvant | | | |
| Phase of Development: | Pilot phase I/II | | Indication: | Human immunodeficiency virus-1 (HIV-1) infection |
| **Study Center(s):** |  | | | |
| Objectives: | **Primary:**  The primary objective of this study is to evaluate the safety of intranasal administration of Vacc-4x with Endocine as adjuvant at three different dose levels.  **Secondary:**   - To evaluate cellular immune response to Vacc-4x in vitro. - To evaluate cellular immune response to Vacc-4x in vivo by DTH. - To evaluate the effect on CD4 counts and viral load (HIV-1 RNA).   **Exploratory:**   - To evaluate the humoral immune responses in mucosal fluids (nose, rectum, vagina (optional)) and in serum. - To evaluate time dependent change in Vacc-4x specific T cell responses. - To evaluate the immune response in urine. | | | |
| Study Design: | A single-blinded, randomized, sequential study. | | | |
| Planned Number of Subjects: | Four groups of patients with 6 patients in each group. Total 24 patients | | | |
| Subject Population: | HIV-infected patients | | | |
| Diagnosis and Main Criteria for Inclusion/ Enrollment: | **Inclusion criteria:**   - Age above 18 years, both genders. - HIV positive at least one year. - Clinically stable on ART for the last 6 months (changes in therapy is allowed as long as the viral load is stable). - Documented viral load (HIV-1 RNA) less than 50 copies/mL for the last six months. - Documented stable CD4 cell count ≥ 400x10^6^/L. - Nadir (lowest ever) CD4 cell count ≥ 200x10^6^/L. - Signed informed consent.   **Exclusion criteria:**   - Reported pre-study AIDS-defining illness within the previous year. - Malignant disease. - On chronic treatment with immunosuppressive therapy. - Unacceptable values of the hematologic and clinical chemistry parameters, as judged by the Principle Investigator (or designee), including creatinine values >1.5x upper limit of normal (ULN), and AST (SGOT), ALT (SGPT) and alkaline phosphatase values >2.5x ULN. - Concurrent chronic active infection such as chronic viral hepatitis B or C or active tuberculosis. - Pregnant or breastfeeding women. - Women of childbearing potential not using reliable and adequate contraceptive methods (defined as: use of oral, implanted, injectable, mechanical or barrier products for the prevention of pregnancy; practicing abstinence; sterile) during the study, or sexually active male patients with partners of childbearing potential unwilling to practice effective contraception during the study. - Current participation in other clinical therapeutic studies. - Incapability of compliance to the treatment protocol, in the opinion of the Investigator. | | | |
| Reference Product: | None | | | |
| Treatment Regimen: | During the Treatment Period, all subjects will remain on their ART and receive four immunizations at weeks 1, 2, 3 and 4 with either :  ***A low dose group***: 80 µg Vacc-4x (20 µg pr. peptide) divided into two administrations;  40 µg Vacc-4x in 150 µl Endocine for each nostril.  ***A medium dose group***: 400 µg Vacc-4x (100 µg pr. peptide) divided into two administrations;  200 µg Vacc-4x in 150 µl Endocine for each nostril.  ***A high dose group***: 1200 µg Vacc-4x (300 µg pr. peptide) divided into two administrations;  600 µg Vacc-4x in 150 µl Endocine for each nostril.  ***A zero dose group***: Only adjuvant divided into two administrations;  150 µl Endocine for each nostril. | | | |
| Duration of Participation: | Screening: 1-4 weeks.  Study period: 8 weeks (4 weeks immunization period + 4 weeks follow-up) | | | |
| Endpoints: | **Safety endpoints:**   - AEs recorded at each scheduled and unscheduled visit - Clinical laboratory evaluations (clinical chemistry, hematology) at screening, week 1,   week 4 and week 8 (Termination).   - Viral load (HIV-1 RNA) at screening, week 1, week 4 and week 8 (Termination). - Physical examinations at screening, week 1, week 4 and week 8 (Termination). - Vital signs (heart rate, blood pressure) at screening, week 1, week 4 and week 8   (Termination).  **Efficacy endpoints:**   - Immune response – T cell immunogenicity: - T cell responses to Vacc-4x at screening and week 1 compared to week 8CD4 counts at screening, week 1, and week 8 (Termination). - DTH (both induration and erythema) at week 8 in terms of observed areas (mm^2^) and also in terms of a positive test, defined as an area ≥10 mm^2^.   **Exploratory endpoints:**   - Humoral immune responses to Vacc-4x in mucosal fluids and in serum. - Time dependent change in Vacc-4x specific T cell responses. - Immune response in urine.   Post-study follow-up (T cell responses in blood and/or mucosal secretions): At 6 months interval Week 28 (pending documented immunogenicity week 8 in Vacc-4x group), Week 52 (pending documented immunogenicity Week 28) and final Week 76 (pending documented immunogenicity Week 52). | | | |

# introduction

## Background information

Human immunodeficiency virus (HIV) infects the CD4 subset of T-cells that are critical for initiating immune responses to infection. The level of CD4 cells in the blood is a marker of a patient’s immunological status. Before the advent of HIV medication, it was clear that sustained immune responses to the HIV capsid protein, p24, were inversely correlated with disease progression (CD4 cell decline) ([Forster, 1987](#Forster1987); [Weber, 1987](#Weber1987)). When CD4 counts are below 200x10^6^/L, immune status is reduced to a level that allows for opportunistic infections. In contrast, long term non-progressor patients showed sustained immune responses to HIV p24. Sustained immune responses to p24 appear to be essential to maintain CD4 counts and thereby restrict disease progression.

Vacc-4x is a peptide-based HIV immunotherapy. The primary objective of Vacc-4x immunotherapy is to strengthen the immune system’s response to HIV p24. The immune response to HIV-1 following immunization with Vacc-4x could overcome the hazards (opportunistic infection, death) associated with subjects on antiretroviral therapy (ART) with insufficient regain of CD4 counts.

ART dramatically reduces the level of virus in circulation in the body, thereby allowing the immune system to focus on the therapeutic vaccine that is administered. ART also allows for the generation of new naïve CD4 cells that can be triggered by the immunotherapy to generate new immune responses to HIV-1. However, recently published results indicate that residual HIV-1 replication may be ongoing despite modern ART ([Dinoso, 2009](#Dinoso2009); [Trono, 2010).](#Trono2010) This may increase the patient’s susceptibility to immune activation and poor immune reconstitution, which both may lead to complicating disease. Adding Vacc-4x immunizations to ART would potentially provide further improved control of virus replication.

Vacc-4x is comprised of peptides that are modified from conserved domains of HIV p24, the major capsid protein of HIV. So far it has been administered intradermally following the administration of granulocyte macrophage colony stimulating factor (GM-CSF), which is used as a local adjuvant. GM‑CSF was chosen because of its ability to facilitate dendritic cell maturation and migration to the lymph nodes for antigen presentation ([Disis, 1996](#Disis1996); [Banchereau, 1998](#Banchereau1998)). Dendritic cells are targeted because they are the most potent antigen presenting cells of the immune system and have the capacity to carry out ‘cross presentation’ during which foreign antigens are presented on both human leukocyte antigen (HLA) classes 1 and 2 (Figure 1).

Figure 1 Simplified Diagram Showing the Two Pathways for Peptide Digestion Leading to Presentation at the Cell Surface


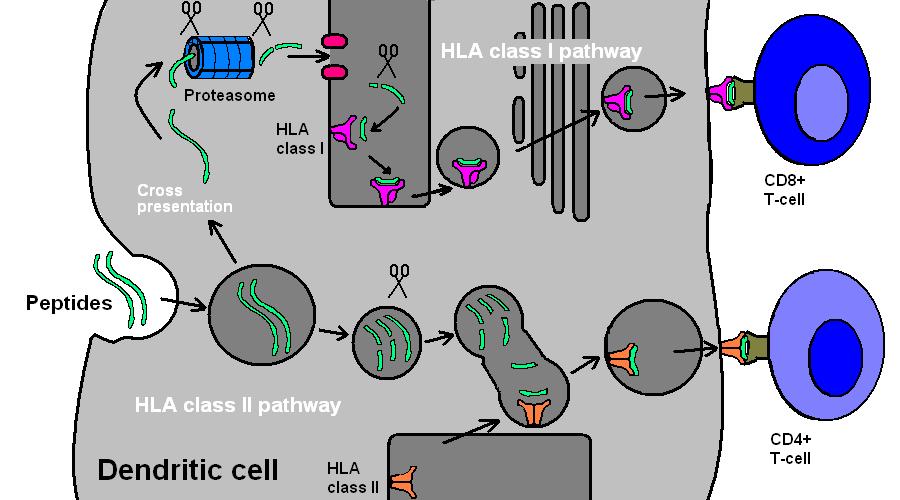


Dendritic cells ingest peptides into small vesicles. If they follow the HLA class II pathway, they are cleaved within the vesicle that subsequently fuses with one carrying the HLA class II molecule. Some of the peptide fragments bind to the HLA class II and reach the cell surface attached to this molecule. The peptide fragment is recognized by a CD4 cell that docks onto the HLA molecule. Dendritic cells have the unique ability to transfer peptides to the HLA class I pathway. Here, the peptides are digested by the proteasome. The peptide fragments are then transported into specialized compartments of the cell where the HLA class I molecules reside. The peptides attach themselves to the HLA class I molecule and are transported to the cell surface. Peptide fragments are recognized by a CD8 cell that docks onto the HLA class I molecule. (From: [Sommerfelt, 2004)](#Sommerfelt2004)

A consequence of dendritic cell stimulation by peptides is the proliferation of both CD4 and CD8 cells. The CD8 cells acquire the ability to recognize infected cells expressing HIV epitopes that are represented in the modified peptides. The Vacc-4x immunotherapy therefore aims to stimulate cell-mediated immunity. Since HIV can persist in quiescent T cells and memory cells ART cannot cure the disease, but must be a life-long treatment. Stimulation of the cell-mediated immunity might help reaching the HIV reservoirs that lie in sites poorly accessed by current ART (such as gut and brain).

## Investigational product

Vacc-4x

Vacc-4x is comprised of peptides that are modified from conserved domains of HIV p24, the major capsid protein of HIV. Prior to the introduction of ART, it became clear that the survival time during HIV-1 infection was related to immune responsiveness to the major capsid protein p24. Sustained immune responses to p24 should maintain CD4 counts and delay disease progression. It has been shown that p24 antigens (not virus-associated) can persist during ART. Increasing levels of p24 antigen while on ART are inversely correlated with CD4 count and disease progression. The Vacc-4x peptides are based on conserved domains of the major capsid protein p24.

## Nonclinical studies

Conclusions from Nonclinical Studies

The results of non-clinical single-dose studies in mice and rats indicate that the dose levels of intravenous Vacc-4x elicited no adverse reactions and that the no effect level was in excess of 3 mg/kg, which constitutes a 500‑fold safety margin over the planned human dose level.

In a rabbit study the effect of Vacc-4x was evaluated in the presence of concomitant GM-CSF, the adjuvant used in the clinical program. Local intradermal reactions such as erythema and edema were noted, however, similar effects were noted in control animals both macroscopically and histologically. These local reactions were slightly more pronounced in the Vacc-4x treated animals. There were no systemic reactions in this study. These data indicate that Vacc-4x has no limiting toxicology in a model that is relevant to the proposed clinical study.

## Clinical Studies

Four clinical studies have been conducted with Vacc-4x; a Phase I clinical study at the University of Bergen, Norway including 11 patients, and a Phase II clinical study at Ullevål University hospital in Oslo, Norway that enrolled 40 chronic HIV‑infected patients ([Åsjö, 2002;](#Åsjö2002) [Kran, 2004](#Kran2004)). In a follow-up of the patients at former Ullevål university hospital 7 years after immunization, most patients who earlier had responded to Vacc-4x now showed detectable CD4 memory cells which could be activated (Report CTN-BI/Vacc-4x/2009/1 2011-02-02). In addition, one phase II study has just been finished, i.e. a randomized, double-blind, multicenter, immunogenicity study of Vacc-4x versus placebo in patients infected with HIV-1 who have maintained an adequate response to ART (EudraCT Number 2007-006302-13). This study included 135 patients. In this study no effects on CD4 counts was seen but there was a favorable effect on viral load.

## Adjuvant

The Endocine adjuvant is a lipid based nano-emulsion consisting of mono-olein and oleic acid in 0,15 M NaOH at pH 8,0. The nanoparticles have a size of less than 100 nm. The appearance of the nanoparticles is a white opalescent liquid with reological characteristics similar to water.

The two lipids are endogenous and upon administration to a mammal they are incorporated into the normal biochemical pathways within the body and metabolised.

Both of the lipids are approved for pharmaceutical use (Ph Eur. 6^th^ Ed. (6:3) 2009).

***Toxicology studies of Endocine:***

Safety testing of Endocine as adjuvant for an influenza vaccine has been performed at Visionar AB in Uppsala, Sweden in order to assess general toxicity of the vaccine formulations. In one study ([Visionar Final Report of Study VP-07-07 (2007](#VisionarReport2007))), it was concluded that the H1N1 antigen used in this study provoked an immunological response which stimulated antibody production against H1N1 virus. The severity and frequency of lesions in adjuvant or adjuvant + virus antigen treated animals were similar to frequency of lesions found in vehicle treated animals. This indicates that the adjuvant or adjuvant + virus antigen did not produce mucosal lesions.

Health records haematology, clinical chemistry and histopathology analyses indicate that the vaccine, after repeated administration, did not cause general toxic reactions in rats.

In a second toxicology study in rats general toxicity of the vaccine formulation was assess ([Visionar Final Report of Study VP-08-35-01 (2009))](#VisionarReport2009). The data assembled show that nasal administration of the adjuvant anionic adjuvant alone or in combination with the A/Solomon Islands/3/06-like IVR-145 (H1N1) whole cell influenza virus antigen produced very few histopathological effects. These can be explained by a normal reaction against immunologically active agents or caused at blood sampling, however. Taking all data collected in this study into consideration it seems as the anionic adjuvant (at concentrations of 0.5 or 2%) alone, or in combination with the A/Solomon Islands/3/06-like IVR-145 (H1N1) whole cell influenza virus antigen, was tolerated by rats.

## Rationale for the Study

Adding Vacc-4x immunizations to ART could potentially reduce virus replication further by removing infected cells and thereby act to slow down the disease progression. Thus, by administering Vacc-4x to subjects who are clinically stable on ART, immune responses against HIV p24 should be boosted leading to improved health status.

In the previous studies with Vacc-4x it has been used as an intra-dermal administration using GM-CSF as an adjuvant. Since intra-dermal administration is dependent on trained health personal it is of interest to test a more simple administration route.

This study is to evaluate safety and immune response (T-cell response) after mucosal delivery (intranasal administration) of three different doses of Vacc-4x using Endocine as adjuvant to patients stable on ART.

### Dose

Vacc-4x

Based on previous studies, 400 µg Vacc-4x (100 µg pr. peptide) and 1.2mg Vacc-4x (300 µg pr peptide) were chosen as the medium and highest dose group. In addition a low dose group of 80 µg Vacc-4x (20 µg pr. peptide) is to be included.

A DTH test (Vacc-4x 400 µg intra-dermal) will be done at week 8.

### Immunization schedule

The same 4 week initiation dosing regimen (weeks 1, 2, 3 and 4) as used in previous studies will be used.

### Use of adjuvant Endocine

Peptide vaccines are poorly immunogenic by themselves. To induce measurable levels of T‑helper cell type 1 (Th1) or type 2 (Th2) immune responses against these peptides, adjuvant is often required.

The Vacc-4x nasal vaccine is prepared by a mixing of the Endocine adjuvant nanoparticles and the Vacc-4 peptides.

## Potential Risks and Benefits

Participants will provide important scientific information that may contribute to improved management of future HIV-positive subjects. Potential benefits for participants include a possible sustained improvement in the immune response to p24 and HIV. All patients will continue their regular ART regimen throughout the study and the study design does not involve scheduled ART interruption. Potential risks include the discomfort and inconvenience associated with the immunizations and the risk of known or unknown side effects of exposure to Vacc-4x given as intranasal administration with Endocine as adjuvant.

After intranasal administration the most common types of adverse events are expected to be related to local irritation of the nasal mucosa.

# Study objectives AND ENDPOINTS

## Study objectives

### Primary objective

The primary objective of this study is to evaluate the safety of intranasal administration of Vacc-4x with Endocine as adjuvant at three different dose levels.

### Secondary objectives

- To evaluate cellular immune responses to Vacc-4x in vitro.
- To evaluate cellular immune responses to Vacc-4x in vivo by DTH in comparison to DTH from unvaccinated patients on effective ART (data from preceding study).
- To evaluate the effect on CD4 counts and viral load (HIV-1 RNA).

### Exploratory objectives

- To evaluate the immune response in mucosal fluids (nose, rectum, vagina (optional)) and in serum.
- To evaluate time dependent change in Vacc-4x specific T cell responses.
- To evaluate the immune response in urine.

## Study endpoints

### Safety endpoints

- AEs recorded at each scheduled and unscheduled visit
- Clinical laboratory evaluations (clinical chemistry, hematology) at screening, week 1, week 4 and week 8 (Termination).
- Viral load (HIV-1 RNA) at screening, week 1, week 4 and week 8 (Termination).
- Physical examinations at screening, week 1, week 4 and week 8 (Termination).
- Vital signs (heart rate, blood pressure) at screening, week 1, week 4, and week 8 (Termination).

### Efficacy endpoints

- Immune response – T cell immunogenicity:

T-cell responses to Vacc-4x at screening and week 1 compared to week 8.

- CD4 counts at screening, week 1, week 4 and week 8 (Termination).
- DTH (both induration and erythema) at week 8 in terms of observed areas (mm^2^) and also in terms of a positive test, defined as an area ≥10 mm^2^.

### Exploratory endpoints

- Humoral immune response to Vacc-4x in mucosal fluids and in serum.
- Time dependent change in Vacc-4x specific T cell responses.
- Immune response in urine.
- Post-study follow-up (T cell responses in blood and/or mucosal secretions): At 6 months interval Week 28 (pending documented immunogenicity week 8), Week 52 (pending documented immunogenicity Week 28) and final Week 76 (pending documented immunogenicity Week 52).

# study design

## Overall study design and flow chart

The study is a single-blinded, sequential design, single centre pilot study in HIV-positive subjects on treatment (ART). Twenty-four subjects will be enrolled into one of four treatment groups.

| **Group** | **Treatment** | **No. of subjects in Europe** |
| --- | --- | --- |
| 1 | 80 µg Vacc-4x + Endocine* | 6 |
| 2 | 400 µg Vacc-4x + Endocine* | 6 |
| 3 | 1.2 mg Vacc-4x + Endocine* | 6 |
| 4 | Endocine* | 6 |

* adjuvant

Subjects who have been HIV-positive and stable on ART for the last 6 months and CD4 cell counts ≥400x 10 ^6^ /L and who meet the other inclusion and exclusion criteria will be eligible for the study. The duration of the main study is 8 weeks plus a screening period of up to 4 weeks. A study flow chart is presented in Table 2.

Table 2 Study Flow Chart

| **PARAMETERS** | **Weeks**  **Day** | **-4** | **1 2 3 4**  **1 8 15 22** | | | | | **8 END**  **50-53 (DTH)** | | |
| --- | --- | --- | --- | --- | --- | --- | --- | --- | --- | --- |
|  | Visits | 1 | 2 3 4 5 | | | | |  | | |
|  |  | Scree-ning | Immunization  period | | | | | | Follow-up  period | |
| Randomization | | x |  |  |  |  |  |  | |  |
| Immunization^a)^  VACC-4x / Endocine | |  | x | x | x | x |  |  | |  |
| DTH-test ^b)^ | |  |  |  |  |  |  |  | | x |
| Adverse event | |  | x | x | x | x |  |  | | x |
| Vital signs/Physical examination^c)^ | | x | x |  |  | x |  |  | | x |
| Blood sample:  general safety ^d)^ | | x | x |  |  | x |  |  | | x |
| Blood sample:  Viral load (HIV-1 RNA), CD4 | | x | x |  |  | x |  |  | | x |
| Blood sample:  T-cell response^e)^ | | X | X | x | x | X |  |  | | X |
| Sampling of mucosal secretions | |  | x |  |  | x |  |  | | x |
| Urine sample | |  | x |  |  |  |  |  | | x |
| Inclusion/Exclusion | | x |  |  |  |  |  |  | |  |
| Informed consent | | x |  |  |  |  |  |  | |  |

1. 150µl to be administered in each nostril
2. DTH skin reaction; to be registered at 48 hours after immunization.
3. Body weight, height (at baseline only), blood pressure, heart rate, concomitant disease, concomitant medication
4. Hb, CRP, WBC (incl. diff), Platelets, Creatinine, Na, K, AST, ALT, LDH, ALP, γ-GT, Albumin, Total Bilirubin, Beta_2_-microglobulin. In addition, the subjects HLA type can be registered.
5. For cryopreservation of PBMC to T cell response assays with optional samples for T cell response kinetics and optional assays on fresh blood samples. X, mandatory assay time points; x, optional time points pending on responsiveness.

During the Treatment Period, all subjects will remain on their ART and receive four immunizations at Weeks 1, 2, 3 and 4, with Vacc‑4x including Endocine as adjuvant.

The study is sequential, meaning that the first five subjects receive either adjuvant only (two patients) or the lowest dose (three patients) 80 µg Vacc-4x together with Endocine (given as 40 µg Vacc-4x as 150µl Endocine solution in each nostril). If the safety evaluation after two weeks is ok, the next dose level will be started, see Figure below:


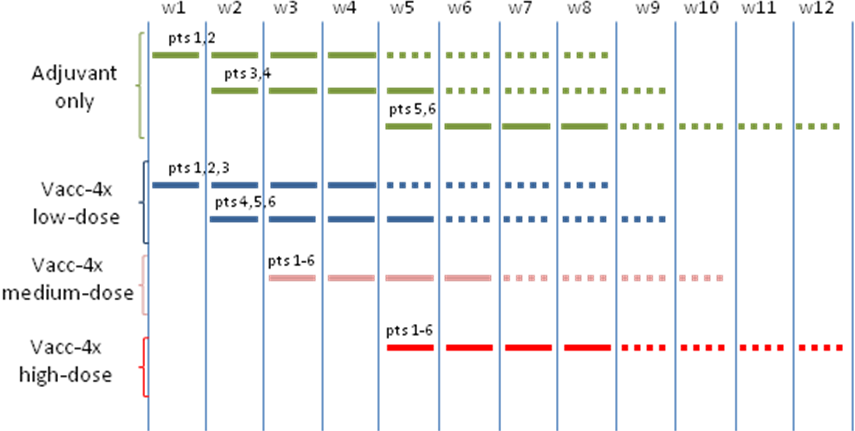


##

## Rationale for study design and treatment regimens

Previous studies have shown that intra-dermal immunization with Vacc-4x using GM-CSF as adjuvant gives an immunological response that is believed to be essential to control HIV better. This study is a pilot study to investigate if intra-nasal administration is a possible administration rout.

# Selection and withdrawal of subjects

## Subject screening, enrollment, and identification log

A subject screening log will be completed at the investigational site for all subjects who were considered for the study but not enrolled and subject screening/enrollment and subject identification logs will be completed for subjects enrolled in the study.

## Inclusion criteria

A subject will be eligible for enrollment in the study if all of the following criteria apply:

- Age above 18 years, both genders.
- HIV positive at least one year.
- Clinically stable on ART for the last 6 months (changes in therapy is allowed as long as the viral load is stable).
- Documented viral load (HIV-1 RNA) less than 50 copies/mL for the last six months.
- Documented stable CD4 cell count ≥ 400x10^6^/L.
- Nadir (lowest ever) CD4 cell count ≥ 200x10^6^/L.
- Signed informed consent.

## Exclusion criteria

A subject will not be eligible for this study if any of the following criteria apply:

- Reported pre-study AIDS-defining illness within the previous year (see Appendix 16.2).
- Malignant disease.
- On chronic treatment with immunosuppressive therapy.
- Unacceptable values of the hematologic and clinical chemistry parameters, as judged by the Principle Investigator (or designee), including creatinine values >1.5x upper limit of normal (ULN), and AST (SGOT), ALT (SGPT) and alkaline phosphatase values >2.5x ULN.
- Concurrent chronic active infection such as chronic viral hepatitis B or C or active tuberculosis.
- Pregnant or breastfeeding women.
- Women of childbearing potential not using reliable and adequate contraceptive methods (defined as: use of oral, implanted, injectable, mechanical or barrier products for the prevention of pregnancy; practicing abstinence; sterile) during the study, or sexually active male patients with partners of childbearing potential unwilling to practice effective contraception during the study.
- Current participation in other clinical therapeutic studies.
- Incapability of compliance to the treatment protocol, in the opinion of the Investigator.

## Withdrawal of subjects

A subject may voluntarily withdraw or be withdrawn from the study at any time for reasons including, but not limited to, the following:

- The subject wishes to withdraw from further participation.
- Continuation in the study would be impracticable or detrimental to the subject’s safety in the opinion of the Investigator.

If a subject is discontinued prematurely from the study, the investigator must provide an explanation in writing and complete the appropriate study discontinuation assessments. If study drug has been administered, the investigator should make every effort to perform all scheduled evaluations, unless the subject has withdrawn consent for study participation.

In the event a subject discontinues treatment prematurely due to an AE, the AE will be followed until it resolves (returns to normal or pretreatment values) or stabilizes, or until it is judged by the investigator to be no longer clinically significant.

Subjects who discontinue participation in the study prior to receiving study drug will be replaced by randomizing an additional subject. Subjects who discontinue participation after receiving study drug will not be replaced.

### Vaccine toxicity management

#### Administration site reactions

The following local reactions will be reported:

- Pain, tenderness, acute swelling or soreness at the application site (nose). The severity of pain or other reactions will be categorized as: None,’ ‘Mild’ (awareness of symptom but easily tolerated), ‘Moderate’ (discomfort enough to cause interference with usual activities), or ‘Severe’ (incapacitating with inability to work or do usual activity)

For administration site reactions judged to be life-threatening or severe, the investigational drug substance suppliers should be notified within 24 hours, and further vaccines should not be given to the subject prior to consultation with the suppliers.

#### Systemic reactions

Systemic reactions will be graded according to the Division of AIDS Table for Grading Severity of Adult and Pediatric Adverse Events (see Appendix 16.1). The investigational drug substance suppliers should be contacted within 24 hours for any systemic Grade 3 or 4 reactions thought definitely, possibly, or probably related to treatment. Further vaccines should not be given subject prior to consultation with the investigational drug suppliers.

# Treatment of Subjects

## Study drug and dosages

### Study drug description

#### Vacc-4x

The Investigational Medicinal Product (IMP), Vacc-4x, consists of four synthetic peptides (Vacc‑10 acetate, Vacc‑11 acetate, Vacc‑12 acetate, and Vacc‑13 acetate), each corresponding to conserved domains on the HIV-1 p24 capsid protein representing the native Gag regions with residues 166-185, 252‑269, 264-284, and 335-354, respectively.

Vacc-4x is manufactured by Bachem AG (Hauptstrasse 144, CH-4416 Bubendorf, Switzerland). Vacc-4x is manufactured in accordance with Good Manufacturing Practice (GMP). Vacc-4x is supplied as sterile vials of freeze-dried white powder. There is no additional ingredient in the product.

Vacc-4x should be mixed with Endocine and administered intranasal as a 150µl in each nostril.

#### Endocine

The Endocine adjuvant is a lipid based nano-emulsion consisting of mono-olein and oleic acid in a physiologically acceptable buffer. The nanoparticles have a size of less than 100 nm. The appearance of the nanoparticles is a white opalescent liquid with reological characteristics similar to water.

Endocine is manufactured by Galenica AB, Malmö, Sweden and is supplied in vials containing 250 µl of the adjuvant.

The final formulation to be administered contains the following ingredients:

| **Substances** | **Generic name** | **CAS number*** |
| --- | --- | --- |
| Mono-oleate | Glyceryl oleate | 97593-29-8 |
| Oleic acid | Oleic acid | 112-80-1 |
| NaOH | Sodium hydroxide | 95077-05-7 |
| Vacc-4 peptides |  | |
| Water | water |  |

* Chemical Abstracts Service. A division of American Chemical Society.

Due to instability of the Vacc-4x peptides, the final vaccine formulation will be mixed in connection with the administration to the subjects participating in the study. By dispensing the Vacc-4x peptides in water and subsequently mixing the peptides with a Endocine suspension prepared in 15 mM NaOH, a stable formulation lasting for at least 8 hours is achieved.

### Dosage and administration of study drug

Vacc-4x is administered intranasal as a mixture of Vacc-4x peptides and the adjuvant Endocine.

The study includes four groups:

***A low dose group***: 80 µg Vacc-4x (20 µg pr. peptide) divided into two administrations;

40 µg Vacc-4x in 150 µl Endocine for each nostril.

***A medium dose group***: 400 µg Vacc-4x (100 µg pr. peptide) divided into two administrations;

200 µg Vacc-4x in 150 µl Endocine for each nostril.

***A high dose group***: 1200 µg Vacc-4x (300 µg pr. peptide) divided into two administrations;

600 µg Vacc-4x in 150 µl Endocine for each nostril.

***A zero dose group***: Only adjuvant divided into two administrations;

150 µl Endocine for each nostril.

The drug will be administered four times with one week interval; at week 1, week 2, week 3 and week 4.

A DTH test (Vacc-4x 400 µg intra-dermal) will be done at week 8.

### Rationale for dosing regimen

The dose and immunization schedule harmonize with the initial phase of previous studies performed with Vacc-4x (Study CTN B-HIV‑2/2001 and Study CT-BI Vacc-4x 2007/1).

### Subject compliance

Clinical staff at the site is to perform the drug administration. They will record the timing of all administrations in the source notes, which will be transcribed onto the case report forms (CRFs). Ongoing compliance with ART will be supported by the site clinicians according to standard site protocols and will not be formally evaluated in this study.

### Overdose and toxicity management

Following each administration, the subject will be required to remain at the clinic for at least 30 min for observation of any immediate reaction to the administration.

There is no experience with human overdose of Vacc-4x even after systemic (intradermal) injections of high dose. The experience in the nonclinical intravenous studies with mice and rats suggests that even if Vacc-4x is inadvertently delivered to the circulation; clinically important toxicity should not be expected.

## Concurrent medications and Non‑drug therapies

Administration of all concomitant medications must be reported in the appropriate section of the CRF along with dosage information, dates of administration, and reasons for use. Trade names (product names) for concomitant medication should be used, if possible. The total daily dose should be recorded whenever possible. Additionally, diagnostic, therapeutic, or surgical procedures performed during the study period must be recorded in the comments section of the corresponding AE report.

### Permitted medications

Any treatment, which is considered necessary for the subject’s welfare (including supportive care) and which will not interfere with the study drug, may be given at the discretion of the investigator.

### Prohibited medications

Subjects are prohibited from concomitant use of any of the following medications:

- Any other immunomodulating therapy
- Chronic corticosteroids, except for asthma inhalers or topical use
- Other investigational drugs, agents, or devices

## Study drug management

### Packaging of study drug

Vacc-4x will be provided as an investigational drug with the appropriate cautionary statements included on the label per local regulatory requirements.

Endocine will be provided as an investigational adjuvant with the appropriate cautionary statements included on the label per local regulatory requirements.

### Labeling of study drug

The vials of Vacc-4x and with Eurocine (adjuvant) will be labeled with the protocol number, expiration date, batch number, and investigational drug substance delivery address. Additional information will, if needed, be included according to local laws and regulations.

### Preparation of study drug

The preparation of study drug; Vacc-4x and Endocine should be performed by the study site pharmacist (or other responsible person). The date (DD/MM/YYYY) and time (XX: XX [24 hours]) of reconstitution should be recorded. The reconstituted product should be used as soon as possible after preparation. Due to instability of the Vacc-4x peptides, the final vaccine formulation will be mixed in connection with the administration to the subjects participating in the study. The stability of the final vaccine formulation has been tested by physiochemical stability testing of the emulsion. No changes were seen during the 12 hour observation period.

Vacc-4x is to be reconstituted with sterile Water for Injection (without preservative), United States Pharmacopoeia (USP) or European Pharmacopoeia (Eur Pharm).

The dose of Vacc-4 peptide is 80 µg, 400 µg and 1200 µg, respectively. After reconstituting, the peptide solution is mixed with the Endocine adjuvant at a 1+1 ratio and subsequently administered to the subject with 150 ul into each nostril (total 300 ul).

### Preparation of DTH-test

DTH skin reactivity testing represents a simple *in vivo* method for monitoring cellular immune responses. The DTH test consists of Vacc-4x with a concentration of 4.0mg/mL. At the pharmacy, a DTH dose is prepared to the final concentration 4.0mg/mL as described in the Pharmacy Manual. A volume of 0.1mL should be drawn into a syringe ready for use. All handling of the product should be in compliance with handling of sterile product for injection. The reconstituted product should be used as soon as possible after preparation, within a maximum of 8 hours. The peptide solution should be stored at 2 to 8^o^C.

### Storage of study drug

Lyophilized Vacc-4x should be stored at 2 to 8^o^C within the expiry date printed on the certificate of analysis. Reconstituted Vacc-4x should be stored at 2 to 8^o^C. Once reconstituted, Vacc-4x is stable for 8 hours.

Endocine adjuvant should be stored at 2 to 8° C. Before mixing with peptide, turn the vial upside down 2-3 times in order to obtain a homogenous emulsion.

### Study drug shipping and handling

Study drugs will be dispatched to a site only after receipt of required documents in accordance with applicable regulatory requirements.

Study drug must be prepared, dispensed and administered according to procedures described herein and in the pharmacy instructions for the study. Only subjects enrolled in the study may receive study drug, in accordance with all applicable regulatory requirements. Only authorized site staff may supply or administer study drug. All study drugs must be stored in a secure area with access limited to the Investigator and authorized site staff and under physical conditions that are consistent with study drug specific requirements.

### Study drug accountability

The pharmacy (or other qualified designee) is responsible for maintaining accurate records of Vacc-4x shipments (receipts) and for recording the use of Vacc-4x on accountability forms. The pharmacy is also responsible for the dispensation and return of the Vacc-4x. Any Vacc‑4x accidentally or deliberately destroyed must be accounted.

Partially used vials must be accounted before they are destroyed at the study center according to local procedures.

Unused vials must be destroyed locally or returned to the investigational drug substance suppliers. At the finalization of the clinical study, the overall numbers of Vacc-4x vials shipped to the study centre and the number destroyed and/or returned must be provided by the pharmacy. An account must be given of any discrepancies that might have occurred during the study.

The pharmacy (or other qualified designee) is responsible for maintaining accurate records of Endocine shipments (receipts) and for recording the use of Endocine on accountability forms.

## Procedures for assigning subjects to treatment groups

24 subjects will be randomly enrolled into one of four treatment groups:

***A low dose group***: 80 µg Vacc-4x (20 µg pr. peptide) divided into two administrations;

40 µg Vacc-4x in 150 µl Endocine for each nostril.

***A medium dose group***: 400 µg Vacc-4x (100 µg pr. peptide) divided into two administrations;

200 µg Vacc-4x in 150 µl Endocine for each nostril.

***A high dose group***: 1200 µg Vacc-4x (300 µg pr. peptide) divided into two administrations;

600 µg Vacc-4x in 150 µl Endocine for each nostril.

***A zero dose group***: Only adjuvant divided into two administrations;

150 µl Endocine for each nostril.

# Study Procedures

## Time and events schedule

The study procedures to be conducted for each subject are divided into the following study phases: Screening (up to 4 weeks), Treatment Period (4 weeks), Follow-up Period (4 weeks).

The procedures to be completed during each of these phases are presented in the Study Flow Chart (Section 4.1, Table 2). Detailed descriptions of the assessments and the definition of study endpoints are provided in Section 8 (Study Assessments and Measurements). Any deviation from study procedures should be noted in the source documents and significant deviations should be reported to the Investigational drug substance suppliers immediately.

## Subject informed consent

Prior to performing any study procedures, the investigator (or his/her designated staff) will obtain written informed consent from the subject as described in Section 12.6.1. After reading the informed consent document and having all questions answered, the subject must give consent in writing. The subject's consent must be confirmed at the time of the subject’s signature by the personally dated signature of the person conducting the informed consent discussions. A copy of the signed consent document must be given to the subject or the subject's legally authorized representative. “Legally authorized representative” means an individual or judicial or other body authorized under applicable law to consent on behalf of a prospective subject to the subject’s participation in the procedure(s) involved in the research. The investigator will retain the original signed consent document. The investigator will not undertake any measures specifically required for the clinical study until valid consent has been obtained.

## Procedures by main study phases

### Pre-treatment period

#### Screening visit (Visit 1) (Week -1 to –4)

Having given consent, subjects will undergo screening assessments to determine whether they are eligible to participate in the study according to the criteria listed in Section 5.2 and Section 5.3. The following evaluations will be performed during screening:

- Review of inclusion/exclusion criteria
- Medical/surgical history (including current disease, pre‑ART CD4 counts, and pre‑ART HIV-1 viral load)
- Physical examination (including height)
- Vital signs (blood pressure, heart rate)
- Clinical chemistry and hematology (hemoglobin, CRP, WBC, platelets, creatinine, Na, K, AST, ALT, LDH, ALP, γ-GT, albumin, total bilirubin, β2-microglobulin).
- Viral load (HIV-1 RNA) andCD4 counts
- T cell responses.
- Concomitant medications and AEs

At the investigator’s discretion, laboratory tests may be ordered through the local laboratory to confirm a subject’s current status regarding chronic active infections or AIDS related illnesses in order to confirm eligibility in the trial if the status is not sufficiently documented in the subject’s medical records.

***Randomization:*** When the results of the screening evaluations are available to confirm eligibility the subjects will be randomized.

### Treatment Period

The treatment period consists of a 4-week period with four weekly administration of study drug, beginning at day 1.

#### Visit 2 (Week 1 - Day 1)

This visit will occur at least five days but not more than 28 days after the screening visit. The following procedures will be performed in the following order (it is important that all blood samples are taken before drug administration):

- Review of concomitant medications and AEs
- Physical examination, including weight
- Vital signs
- Clinical chemistry and hematology and optional HLA type
- Viral load (HIV-1 RNA) and CD4 counts
- T-cell responses
- Collection of mucosal sections
- Collection of urine
- Administration of study drugs via intranasal administration

The subject should remain in the clinic for 30 min after the administration of study drug to be monitored for adverse reactions to the immunization.

#### Visit 3 (Week 2 – Day 8(±1))

The following procedures will be performed in the following order:

- Review of concomitant medications and AEs
- Administration of study drugs via intranasal administration
- T-cell responses (optional)

The subject should remain in the clinic for 30 min after the administration of study drug to be monitored for adverse reactions to the immunization.

#### Visit 4 (Week 3 – Day 15 (±1))

The following procedures will be performed in the following order:

- Review of concomitant medications and AEs
- Administration of study drugs via intradermal injection
- T-cell responses (optional)

The subject should remain in the clinic for 30 min after the administration of study drug to be monitored for adverse reactions to the immunization.

#### Visit 5 (Week 4 – Day 22 (±1))

The following procedures will be performed in the following order (it is important that all blood samples are taken before drug administration):

- Review of concomitant medications and AEs
- Physical examination, including weight
- Vital signs
- Clinical chemistry and hematology
- Viral load (HIV-1 RNA) and CD4 counts
- T-cell responses
- Collection of mucosal secretions
- Administration of study drugs via intranasal administration

The subject should remain in the clinic for 30 min after the administration of study drug to be monitored for adverse reactions to the immunization.

### Follow‑up period

The follow-up period consists of a 4-week period following the Week 4 visit.

#### Visit 6 (Week 8 – Day 50 (±3)) – END OF STUDY

The following assessments will be performed:

- Review of concomitant medications and AEs
- Physical examination, including weight
- Vital signs
- Clinical chemistry and hematology
- Viral load (HIV-1 RNA) and CD4 counts
- T-cell responses
- Collection of secret from nose and rectum
- Collection of urine
- DTH test (immunization to occur on designated day; test to be evaluated 48 hours after immunization)

The subject should remain in the clinic for 30 min after the administration of the DTH test to be monitored for adverse reactions to the DTH test.

#### Visits 7 (Week 28), 8 (Week 52) and 9 (Week 76) (exploratory endpoints)

Post-study follow-up (T cell responses in blood and/or mucosal secretions): At 6 months interval Week 28 (pending documented immunogenicity week 8 in Vacc-4x group), Week 52 (pending documented immunogenicity Week 28 in Vacc-4x group) and final Week 76 (pending documented immunogenicity Week 52 in Vacc-4x group):

The following assessments may be performed:

- T-cell responses
- Collection of secret from nose and rectum
- Collection of urine
- Viral load (HIV-1 RNA) and CD4 counts

## Early withdrawal visit

Subjects may elect to discontinue from the study (i.e., withdraw consent for additional study follow-up) at any time. Every effort will be made to have the subject return to the clinic for a terminal evaluation.

## Subject discontinuation (lost to follow‑up)

In the event that a subject elects not to return to the clinic for the final study visit (Termination), the investigator must make every effort to contact the subject to review all AEs and collect study data. In the event that a subject drops out of the study at any time, the reason for discontinuation must be fully documented in the source documents and the CRF. The site personnel will document the AEs and any other assessments in the source documents and will make every effort to complete all required end of study assessments.

# Study Assessments and Measurements

## Demographic and screening assessments

The following demographic information and baseline characteristics will be collected at the screening visit:

- Date of birth
- Gender
- Ethnic origin
- Physical examination including height (measured in cm). Height measurement may also be obtained at Visit 2.
- Medical/surgical history (including current disease, pre‑ART CD4 counts, and pre‑ART HIV-1 viral load)
- Medication history
- HLA type (will be tested based on blood sample taken at visit 2)
- Vital signs (including blood pressure and heart rate)
- HIV-1 disease status as measured by viral load and CD4 counts
- ART medications and confirmation of clinically stable on the current ART regimen
- Clinical chemistry and hematology

## Efficacy assessments

### Delayed type hypersensitivity test administration

DTH testing will be performed at the last visit (Week 8). An injection of 0.1mL of a 4.0mg/mL Vacc‑4x should be administered intradermally at the front of left arm. The DTH test should be performed by the investigator, study nurse, or other clinician experienced in the techniques of intradermal injection. The DTH test results will be evaluated 48 hours after the tests are performed. For each DTH test, evaluation of the injection site at 48 hours will include palpable skin induration and skin erythema. A positive DTH test is defined as an area ≥10mm^2^ (either induration or erythema). Study personnel will record DTH results in the subject’s source documents and transcribe them into the CRF. For subjects whose DTH reactions are measured by someone other than study personnel, the investigator is responsible for collecting and recording the result; use of a prepared form completed by the alternative evaluator at the time of the reading is strongly recommended and should be filed with the subject’s source documents.

### CD4 counts

A blood sample (approximately 5mL) will be collected in tubes containing EDTA at Screening and Weeks 1, 4, and 8 (Termination). Tests will be performed at the local hospital laboratory.

### T-cell response

Blood samples (approximately 30 mL) will be drawn and the peripheral blood mononuclear cells (PBMC) separated. The PBMC will be frozen at -150°C, optionally will a fraction of fresh PBMC be tested. T-cell response to the Vacc-4x peptides will be measured in vitro by T cell activation assays (proliferation, cytokines) using flow cytometry (see assay [(Kvale, 2005)](#Kvale2005) and/or ELISPOT. Optionally, proinflammatory and inhibitory cytokines as well as tests for cytotoxicity (degranulation, secretion of Granzyme B or equivalents). T-cell proliferative responses will be compared with data reported in the preceding study CTN B-HIV-2/2002.

### Exploratory analyses

Time dependent change in Vacc-4x specific T cell responses: Provided that changes in T cell-responses to Vacc-4x can be achieved between study baseline and end-of-study by this new immunization methodology, as detected by new enhanced proliferative response and fraction of Vacc-4x-specific T cells in blood (activation-related cytokines such as IFN-γ, IL-2, TNF-α as well as degranulating CD8+ T cells –producing), explorative analysis of the effects of each immunization will be of considerable interest. At present, no optimal vaccination schedule for T cell-dependent has been described in this new area of research.

Humoral responses to Vacc-4x in blood, mucosal sites and urine: Provided that changes in humoral responses in blood to Vacc-4x can be achieved between study baseline and end-of-study, exploratory analysis of samples from blood, mucosal fluid from rectum, vagina (optional) and urine may reveal the dose- and vaccination schedule-dependence in the development of such antibody responses.

## Safety assessments

### Medical history

A detailed medical history, including a review of all body systems, pre‑ART CD4 counts, and pre‑ART HIV-1 viral load, will be obtained from each subject during the screening visit. Each subject will be questioned by the nurse/study coordinator/investigator/sub-investigator regarding significant past medical history, medical history related to concurrent diseases, concurrent drug use, and drug allergies.

### Physical examination

Physical examination will be performed at screening, week 1, week 4 and week 8 (end of study). Physical examination will include height (only at screening or week 1) and weight and evaluation of the subject’s medical condition. Any new or worsening of medical condition compared to the pretreatment condition (pre-dose on Day 1) should be recorded as an AE on the CRF.

### Vital signs

At screening, week 1, week 4 and week 8 (end of study) vital signs (heart rate, systolic and diastolic blood pressure) will be measured prior to administration of study drug (if applicable) with the subject in the sitting position. Vital signs will be measured after the subject has been sitting at rest for 5 minutes.

### Clinical laboratory tests

Blood samples will be collected (approximately 15 mL) at screening, week 1, week 4 and week 8 (end of study) to measure the following clinical chemistry and hematology parameters: It is important that blood samples are taken before drug administration. Tests will be performed at the local hospital laboratory.

**Chemistry:** Aspartate Transaminase (AST), Alanine Transaminase (ALT), Creatinine, Na, K, C-reactive Protein (CRP), Alkaline phosphatase (ALP), Lactate Dehydrogenase (LD), Albumin, Total Bilirubin, β2-microglobulin, γ-Glutamyl Transferase.

**Hematology:** Hemoglobin, White blood cell count (WBC) with differential, Platelets.

### HIV viral load (HIV-1 RNA)

A blood sample (approximately 10mL) will be collected in tubes containing EDTA at screening and week 1, week 4 and week 8 (end of study). Tests will be performed at the local hospital laboratory.

# Adverse Event Management

## Non‑serious and serious adverse events

The investigator is responsible for the detection and documentation of events meeting the criteria and definition of an AE or SAE as provided in this protocol (see below). All AEs and SAEs that occur from the time written informed consent is obtained until completion of the study, week 8, will be entered on the appropriate CRF page (see Section 9.1.4). Information to be collected includes the description, date and time of onset, intensity, duration, causality, and outcome of the event. Even if the AE is assessed by the investigator as not reasonably attributable to study drug, its occurrence must be recorded in the source documents and reported on the CRF.

Periodically during the study, after the subject has had an opportunity to spontaneously mention any problems, the investigator should inquire about the occurrence of AEs. The following are examples of open‑ended questions that may be used to obtain this information:

- “How are you feeling?”
- “Have you had any medical problems since your last visit/assessment?”
- “Have you taken any new medicines, other than those given to you in this study, since your last visit/assessment?”

### Definition of an adverse event

An AE is any untoward medical occurrence in a subject administered a pharmaceutical product that does not necessarily have a causal relationship with this treatment.

An AE can therefore be any unfavorable and unintended sign (including an abnormal laboratory finding, for example), symptom, or disease temporally associated with the use of a medicinal product, whether or not considered related to the medicinal product.

Examples of AEs include:

- Exacerbation of a pre‑existing illness following the start of the study
- Increase in frequency or intensity of a pre‑existing episodic event or condition
- Condition detected or diagnosed after study drug administration even though it may have been present prior to the start of the study

An AE **does** **not** include:

- Medical or surgical procedure (e.g., surgery, endoscopy, tooth extraction, transfusion); the condition that leads to the procedure is an AE
- Day to day fluctuations of pre‑existing disease or conditions present or detected at the start of the study that do not worsen
- Situations where an untoward medical occurrence has not occurred (e.g., hospitalizations for cosmetic elective surgery, social and/or convenience admissions)
- The disease or disorder being studied or sign or symptom associated with the disease or disorder unless more severe than expected for the subject’s condition
- Overdose of either study drug or concurrent medication without any signs or symptoms

### Definition of a serious adverse event

An SAE is any untoward medical occurrence that at any dose:

- Results in death
- Is life-threatening

*Note:* The term “life‑threatening” in the definition of “serious” refers to an event in which the subject was at risk of death at the time of the event; it does not refer to an event which hypothetically might have caused death if it were more severe.

- Requires inpatient hospitalization or prolongation of existing hospitalization

*Note:* Complications that occur during hospitalization are AEs. If a complication prolongs hospitalization or fulfills any other serious criteria, the event is serious. Hospitalization for elective treatment of a pre‑existing condition that did not worsen from the pretreatment assessment is not considered to be an AE.

- Results in persistent or significant disability/incapacity

*Note:* The term disability means a substantial disruption of a person’s ability to conduct normal life functions. This definition is not intended to include experiences of relatively minor medical significance such as uncomplicated headache, nausea, vomiting, diarrhea, influenza, or accidental trauma (i.e., sprained ankle) that may interfere or prevent everyday life functions but do not constitute a substantial disruption.

- Is a congenital anomaly/birth defect
- Important medical events that may not be immediately life‑threatening or result in death or hospitalization but may jeopardize the subject or may require intervention to prevent one of the outcomes listed in the definition above should also usually be considered serious.

*Note:* Examples of such events are intensive treatment in an emergency room or at home for allergic bronchospasm, blood dyscrasias, or convulsions that do not result in hospitalization; or development of drug dependency or drug abuse.

### Clinical laboratory abnormalities and other abnormal assessments

Laboratory abnormalities are usually not recorded as AEs or SAEs. All abnormal laboratory values judged to be at least possibly drug related or clinically significant abnormal laboratory results of any causality not associated with the disease under study must be repeated. The investigator will exercise medical judgment in deciding whether abnormal laboratory values are clinically significant. In some cases, significant changes within the range of normal will require similar judgment by the Investigator.

Clinically significant abnormal laboratory values should not be listed on the AE page of the CRF unless clinical signs or symptoms are present. If an abnormal laboratory value or assessment is clearly related to a medically defined diagnosis or syndrome, the diagnosis or syndrome will be recorded on the AE page, not the individual laboratory values.

All clinically significant abnormal laboratory results or assessments will be followed until they resolve (return to normal or pretreatment values) or stabilize, or until they are judged by the investigator to be no longer clinically significant.

### Recording of adverse events and serious adverse events

From the point of signing the informed consent form (ICF) to the first administration of study medication, any worsening of an existing or new medical condition must be recorded on the Medical History CRF page. Following administration of study medication up to the week 8 (Termination), any worsening of an existing or new medical condition must be recorded as an AE on the CRF. The investigator should review all documentation (e.g., hospital progress notes, laboratory, and diagnostic reports) relative to the event being reported. The investigator will then record all relevant information regarding an AE/SAE on the appropriate CRF page. The investigator will evaluate AEs using the following guidelines:

- Description of event (if the event consists of a cluster of signs and symptoms, a diagnosis should be recorded [e.g., flu syndrome] rather than each sign and symptom)
- Onset date and time
- Stop date and time
- Intensity should be described according to the Division of AIDS Table for Grading Severity of Adult and Pediatric Adverse Events, December 2004 (Appendix 16.1) as MILD (Grade 1), MODERATE (Grade 2), SEVERE (Grade 3), or life threatening (Grade 4). Intensity is defined as one of the following:
- Mild: transient or mild discomfort; no limitation in activity; no medical intervention/therapy required
- Moderate: mild to moderate limitation in activity- some assistance may be needed; no or minimal medical intervention/therapy required
- Severe: marked limitation in activity, some assistance usually required; medical intervention/therapy required, hospitalizations possible
- Life-threatening: extreme limitation in activity, significant assistance required; significant medical intervention/therapy required; hospitalization or hospice care probable

It is important to distinguish between SAEs and severe AEs. Severity is a measure of intensity, whereas seriousness is defined by the criteria provided in Section 9.1.2. An AE of severe intensity need not necessarily be considered serious. For example, a migraine headache that incapacitates a subject for many hours may be considered a severe AE, whereas a stroke that results in a limited degree of disability may be considered mild, but should be reported as an SAE. If any parameter within a record of an Adverse Event changes, a fresh record should be made in the CRF. For example, but not limited to, where the severity of the AE increases, the AE should be recorded as resolved at the existing severity level and re-entered into the CRF with a new start date reflecting the first date of the increased severity.

- Seriousness (see definition of an SAE, Section 9.1.2)

The investigator must record whether or not the AE meets the definition of serious. If the event is serious, the Investigator must complete an SAE Report Form.

- Relationship to study drug

The investigator must make a causality assessment for all AEs and must decide whether there is a reasonable possibility that the AE may have been caused by the study drug. If there is any valid reason for suspecting that there is a causal relationship between the AE and the study drug, the AE should be judged “possibly related,” “probably related,” or “definitely related” to study drug. Unless an AE can be excluded from causality, it must be judged “possibly related” to study drug rather than “not related” to study drug.

- Outcome

Outcome of AEs should be recorded as resolved, resolved with sequelae, not resolved, or fatal based on the status of the AE at discontinuation from the study. If an AE is not resolved at the time of discontinuation, the AE should be followed until it is resolved (returns to normal or pretreatment values) or stabilized, or until it is judged by the Investigator to be no longer clinically significant.

- Action taken (none, required treatment, reduction of dose, or permanent discontinuation of study drug). *All applicable actions taken should be recorded.*

### Follow-up of adverse events and serious adverse events

All AEs and SAEs must be followed until they are resolved (return to normal or pretreatment values) or stabilized, or until they are judged by the Investigator to be no longer clinically significant. Supplemental measurements and/or evaluations may be necessary to fully investigate the nature and/or causality of an AE or SAE. This may include additional laboratory tests, diagnostic procedures, or consultation with other healthcare professionals. If the subject dies, any findings (including histopathology) must be provided to the Investigational drug substance suppliers.

### Reporting of all serious adverse events and any adverse events resulting in study discontinuation

The investigator will promptly report all SAEs to the regulatory and ethical agencies.

Any SAE, regardless of expectedness or causality, must be reported by telephone and by faxing the completed SAE Report Form to the investigational drug substance suppliers within 24 hours of the investigator or any other site personnel’s knowledge of the event. In addition, any AE resulting in permanent study discontinuation for a subject, even if not serious, must be reported immediately to the Investigational drug substance suppliers. An updated SAE Report Form should be forwarded to the investigational drug substance suppliers within 24 hours of receipt of the new/updated information.

Table 3 Serious Adverse Event Reporting Requirements

|  | INITIAL REPORTS | **FOLLOW‑UP REPORTS** |
| --- | --- | --- |
| REPORTING REQUIREMENTS | **24 hours**  Telephone and fully completed SAE Report Form to the Investigational drug substance suppliers | **24 hours**  Updated SAE Report Form to the Investigational drug substance suppliers |

For investigational drugs, an AE is judged expected if its description agrees in nature, severity, frequency, and specificity with the description of events in the current Investigator’s Brochure. For marketed drugs, an AE is judged expected if it is listed in the current package insert or the current product’s packaging materials for the country concerned.

### Post-study adverse events or serious adverse events

Investigators are not obligated to actively seek AEs or SAEs after the follow-up period. However, the investigator should promptly notify the investigational drug substance suppliers if the investigator learns of any SAE or death of a study subject within 30 days after a subject has been discontinued from the study, and such event(s) is (are) reasonably related to the study drug.

Investigators should promptly notify the investigational drug substance suppliers if they become aware of a former study participant who is one of the parents of a subsequently conceived child with a congenital anomaly.

## Overdose

An overdose is not recorded as an AE unless symptoms of the overdose occur, in which case the symptoms are recorded as AEs and attributed to the (over)dose of study drug.

# data collectionData Collection

## Data collection

Subject data will be collected on CRFs and source documents at the clinical site. Prior to the start of the study, the investigator will complete a site personnel log showing the signatures and handwritten initials of all individuals who are authorized to make or change entries on CRFs. All required data are to be recorded on the CRF.

Completed CRFs will be reviewed by the study monitor to ensure completeness and consistency. The study monitor will subject all CRF data to 100% source verification and audit to ensure adequate quality control and assurance of subject data. Any discrepancies found during the CRF review are to be clarified by the investigator.

The investigator must sign and date a declaration on the CRF attesting to his/her responsibility for the quality of all data recorded, and that the data represents a complete and accurate record of each subject’s participation in the study. The original completed CRF will be returned to the investigational drug substance suppliers, and a copy will be retained by the investigator.

## Correcting the case report forms

Errors must be corrected by drawing a single line through the incorrect entry and by writing the new value as close to the original as possible. The correction must then be and dated by an authorized individual.

## Data processing

The completed original CRF will be signed and dated by the investigator and transmitted to the investigational drug substance suppliers (or designee) after each subject completes study participation. A duplicate signed copy of the CRF and a record of the laboratory data for each subject will be retained at the site.

A database will be constructed from the CRFs and the data will be validated both manually and electronically. Clarification of data will be requested from the study site as required.

# sTATISTICal methods and planned analyses

This is a single blinded randomized pilot study designed to investigate the safety and efficacy of intranasal administration of three different dose-levels of Vacc-4x using Endocine as an adjuvant.

## Determination of sample size

It is decided that 6 subjects should be included in each of the four study arms. As this is a pilot study, the sample size has primarily been determined to provide descriptive statistics for the safety and efficacy parameters.

## Randomization

Subjects who meet the entry criteria will be randomized into one of the four study arms by using SAS version 9.2.

## Populations to be analyzed

The following populations will be used in the analysis:

Intention to treat (ITT): Consistent with the intent-to-treat principle, all subjects receiving treatment will be included in the safety and efficacy analyses (full analysis population as defined by ICH-E9).

Per protocol (PP): All ITT subjects who receive the full immunization and who do not incur any major protocol violations that would challenge the validity of their data will be included in an additional analysis of main the efficacy parameters.

## Handling of missing data

Missing data will not be imputed and all summary statistics will be reported based upon observed data.

## Subject accountability

The study disposition of all randomized study subjects will be summarized. Subjects who discontinued study drug prematurely or withdrew from the study will also be summarized and listed with a description of the reason for early termination/withdrawal.

### Treatment compliance

Subject compliance with study drug dosing will be assessed by the study personnel and compliance will be summarized using descriptive statistics.

## Statistical methods

Analyses will be based upon an intent-to-treat approach. In general continuous variables will be described using number of observations (N), mean, median, standard deviation (SD), minimum (min), maximum (max), Categorical data will be presented using counts (N) and percentages (%). Subjects without a baseline or a post-baseline assessment for a particular endpoint will not be included in the analysis of change from baseline for that endpoint. All statistical analyses will be performed using SAS Software Version 9.2 or higher. P-values will be reported as 2-sided p-values. All confidence intervals will be reported using traditional 95% two-sided intervals. No adjustment for multiple comparisons will be made.

### Analysis of demographic and subject characteristics

Demographic and subject characteristics will be summarized using descriptive statistics.

### Safety analysis

The primary objective of this study is to investigate the safety of intra-nasal administration of three different dose-levels of Vacc-4x using Endocine as an adjuvant.

#### Adverse events

For presentation, AE verbatim text will be coded into a Medical Dictionary for Regulatory Activities Terminology (MedDRA) term, and classified by System Organ Class (SOC) and preferred term prior to analysis. AEs will then be summarized and grouped by SOC and preferred term. AEs with onset on or after the start of study medication are considered treatment emergent. AEs reported prior to treatment administration will be reported separately from treatment emergent AEs. A cross reference listing, which will relate SOC, preferred term, and verbatim text will be provided.

In addition, summaries will be provided by incidence, severity (mild, moderate, severe), and relationship to study medication (Related, Unrelated). "Possibly Related," "Probably Related," and "Definitely Related" will be grouped as “Related.”

SAEs and AEs leading to discontinuation will be summarized and listed.

#### Vital signs and laboratory parameters

For vital signs, the observed data at baseline and change from baseline for each measurement day will be summarized with descriptive statistics.

Summary statistics for actual values and for change from baseline will be tabulated for laboratory results by scheduled visit. Subjects with laboratory values outside of the normal reference range at any post-baseline assessment will be summarized. Shifts from baseline laboratory values (to worst severity per subject) will be tabulated.

#### Physical examinations

Simple descriptive statistics will be used to summarize the physical examination findings by visit.

#### Concurrent medications

A summary of all concomitant medications taken during the course of the study will be presented in tabular form by therapeutic drug class and generic drug name using the World Health Organization (WHO) Drug classification.

### Efficacy analysis

The secondary objectives of the study are to evaluate immune response measured by T cell immunogenicity (ELISPOT and proliferation assays), by DTH and to evaluate CD4 and viral load (HIV-1 RNA).

#### Efficacy endpoints

The efficacy endpoints will be summarized using descriptive statistics. Absolute changes in CD4 counts between study start (mean of screening and week 1 [Baseline] values) and Week 8 will be calculated. Both the values registered at week 8 and the changes from baseline will be compared among the treatment arms using analysis of covariance (ANCOVA) including the baseline value in the model. Additionally, the changes over time will be explored (e.g., investigate if the treatment effect continues to increase over time) using linear models taking into account that repeated measures are taken from each subject. Given the small sample size, the focus of these will be primarily to describe the changes associated with the different Vacc-4x doses together with Endocine relative to changes that would be seen with Endocine only.

A number of secondary endpoints have been defined. These endpoints are a mixture of continuous and binary endpoints. Continuous endpoints will analyze using the same approach as described above. Binary endpoints will be summarized using the Cochran-Mantel-Haenszel test and the normal approximation to the binomial distribution.

# study management and ethical and regulatory requirements

## Regulatory approval and Good Clinical Practice

This study will be conducted in accordance with GCP requirements described in the current revision of International Conference on Harmonization of Technical Requirements of Pharmaceuticals for Human Use (ICH) Guidelines and all applicable regulations. Compliance with these regulations and guidelines also constitutes compliance with the ethical principles originating from the Declaration of Helsinki. This study will also be carried out in accordance with local legal requirements.

Before the first subject is enrolled in the study, all ethical and legal requirements must be met.

## Deviations from the protocol and protocol amendments

A copy of the approved protocol and the curriculum vitae of the investigator, together with other required documentation, will be filed with the appropriate regulatory authorities. If it is necessary to amend the protocol during the study, proper notification will be made to the regulatory authorities and ECs in the form of a protocol amendment. Any protocol or other deviations that occur during the study will be documented. Depending on the nature of the deviation, this may be reported to the appropriate regulatory authority.

Once the study has started, amendments should be made only in exceptional cases. The changes then become part of the study protocol.

## Discontinuation of study

The PI or investigational drug substance suppliers reserves the right to discontinue the study for any reason at any time. The study may be stopped at any time if, in the opinion of the investigator or investigational drug substance suppliers, safety data suggest that the medical safety of subjects is being compromised.

## End of study

The data collection period is through week 8 (Study End).

## Study records retention and direct access to source documents

The investigator must retain essential documents until notified by the investigational drug substance suppliers. No study document will be destroyed without prior written agreement between the investigational drug substance suppliers and the PI. Should the PI wish to assign the study records to another party or move them to another location, written agreement must be obtained from the investigational drug substance suppliers.

The investigator must maintain a copy of all data collected for each subject treated (including CRF and source data). In order to assure the accuracy of data collected in the CRF, it is mandatory that representatives of the investigational drug substance suppliers as well as representatives of regulatory authorities and ECs have direct access to original source documents (e.g., subject records, subject charts, laboratory reports). During the review of these documents, the anonymity of the subject will be respected with strict adherence to professional standards of confidentiality.

The investigational drug substance suppliers reserves the right to terminate the study for refusal of the PI to supply source documentation of work performed in this clinical study.

The following includes, but is not limited to, the records that must be retained by the investigator:

- Signed informed consent documents for all subjects
- Subject identification code list, screening log (if applicable), and enrollment log
- Record of all relevant communications between the Investigator and the EC
- Composition of the EC
- Record of all relevant communications between the investigator and the investigational drug substance suppliers (or designee)
- List of sub‑investigators and other appropriately qualified persons to whom the investigator has delegated significant study related duties, together with their roles in the study and their signatures
- Copies of CRFs and of documentation of corrections for all subjects
- Drug accountability records
- All other source documents (subject records, hospital records, laboratory records, etc.)

## Investigator responsibilities

### Subject information and informed consent

Before being admitted to the clinical study, all subjects (and their caregivers, if applicable) must consent in writing to participate. An informed consent form (ICF) and subject information sheet (EU subjects only) will be given to each subject as appropriate, which will contain all regulatory required elements, all ICH‑required elements, and appropriate data protection information, when applicable, in language that is understandable to the subject.

The process of obtaining the informed consent will be in compliance with all regulations, ICH requirements, and local laws.

The investigator (or designee) will review the study with each subject and caregiver. The review will include the nature, scope, procedures, and possible consequences of the subject's participation in the study. The consent and review must be in a form understandable to the subject. The investigator (or designee) and the subject must both sign and date the ICF after review and before the subject can participate in the study. The subject will receive a copy of the signed and dated form, and the original will be retained in the site study files. The investigator (or designee) must emphasize to the subject that study participation is entirely voluntary and that consent regarding study participation may be withdrawn at any time without penalty or loss of benefits to which the subject is otherwise entitled.

If the ICF is amended during the study, the investigator must follow all applicable regulatory requirements pertaining to approval of the amended ICF by the IEC. The site must use the amended consent form for all new patients and repeat the consent process with the amended ICF for any ongoing patients.

### Independent ethics committee approval and other institutional requirements

Before the start of the study, the study protocol, informed consent document, and any other appropriate documents will be submitted to the EC. Per institutional/local regulatory requirements, the study protocol and any other appropriate documents will be submitted to scientific committees for approval.

The investigator will forward to the investigational drug substance suppliers (or designee) a copy of the ECs approval of this protocol, amendments, informed consent form and any changes to the informed consent, and any other submitted documentation that requires approval. The investigator will also keep documentation of study approval by internal scientific committees per institutional/local regulatory requirements.

Study drug can only be supplied to the PI after documentation of all ethical and legal requirements for starting the study has been received by the investigational drug substance suppliers.

### Curriculum vitae

The PI must provide the investigational drug substance suppliers with current curricula vitae for herself/himself and each sub‑investigator. A copy of the current license for each clinician, where issued, must also be provided.

### Laboratory certification and normal values

The PI will also provide the investigational drug substance suppliers with the name and location of the clinical laboratory used for laboratory tests and a signed/dated list of the normal values for all laboratory tests required by protocol.

### Delegation of investigator responsibilities

The investigator should ensure that all persons assisting with the study are adequately informed about the protocol, any amendments to the protocol, the study drug, and their study related duties and functions. The investigator should maintain a list of sub‑investigators and other appropriately qualified persons to whom he or she has delegated significant study related duties.

### Liability and insurance

Liability and insurance provisions for this study are provided by “Legemiddelansvarsforeningen” by Dag Kvale.

## Study monitoring and auditing

In accordance with applicable regulations, GCP, and the procedures of the investigational drug substance suppliers (or designee), the study monitor will periodically contact the site and conduct on‑site visits. The extent, nature, and frequency of on‑site visits will be based on study complexity, enrollment rate, and data quality at the site. Through frequent communications (e.g., letter, e‑mail, telephone), the study monitor will ensure that the investigation is conducted according to protocol and regulatory requirements.

During these contacts, the monitoring activities will include:

- Checking and assessing the progress of the study.
- Reviewing study data collected to date for completeness and accuracy.
- Conducting source document verification by reviewing each subject’s CRF against source documents (e.g., medical records, subject diaries, ICF, laboratory result reports, raw data collection forms).
- Identifying any issues and addressing resolutions.

These activities will be done in order to verify that the:

- Data are authentic, accurate, and complete.
- Safety and rights of the subjects are being protected.
- Study is conducted in accordance with the currently approved protocol (and any amendments), GCP, and all applicable regulatory requirements.

The investigator will allow the study monitor direct access to all relevant documents, and allocate his/her time and the time of his/her staff to the study monitor to discuss findings and any relevant issues.

In addition to contacts during the study, the study monitor will contact the site prior to the start of the study to discuss the protocol and data collection procedures with site personnel.

At study closure, study monitors will conduct all activities as indicated in Section 12.9.

## Quality assurance

At its discretion, the investigational drug substance suppliers (or designee) may conduct a quality assurance audit of this study. Auditing procedures of the investigational drug substance suppliers (or designee) will be followed in order to comply with GCP guidelines and ensure acceptability of the study data for registration purposes. If such an audit occurs, the investigator will give the auditor direct access to all relevant documents, and will allocate his/her time and the time of his/her staff to the auditor as may be required to discuss findings and any relevant issues.

In addition, regulatory authorities may conduct an inspection of this study. If such an inspection occurs, the investigator will allow the inspector direct access to all source documents, CRFs, and other study documentation for source data check and/or on‑site audit inspection. The investigator must allocate his/her time and the time of his/her staff to the inspector to discuss findings of any relevant issues.

## Study termination and site closure

Upon completion of the study, the following activities, when applicable, must be conducted by the study monitor in conjunction with the investigator, as appropriate:

- Copy of all study data to the investigational drug substance suppliers.
- Data clarifications and/or resolutions.
- Accounting, reconciliation, and final disposition of used and unused study drug.
- Review of site study records for completeness.

In addition, the investigational drug substance suppliers reserve the right to temporarily suspend or prematurely terminate this study for any reason.

If the study is suspended or terminated for safety reason(s), the investigational drug substance suppliers will promptly inform the investigator, and will also inform the regulatory authorities of the suspension or termination of the study and the reason(s) for the action. The investigator is responsible for promptly informing the EC, and providing the reason(s) for the suspension or termination of the study.

If the study is prematurely terminated, all study data must be returned to the investigational drug substance suppliers. In addition, the site must conduct final disposition of all unused study drugs in accordance with the investigational drug substance suppliers procedures for the study.

## Site termination

The investigational drug substance suppliers may at any time, at its sole discretion, terminate the study site for various reasons, including, without limitation, the following:

- Failure of the investigator to enroll subjects into the study at a reasonable rate.
- Failure of the investigator to comply with applicable laws and/or pertinent regulations.
- Submission of knowingly false information from the research facility to the investigational drug substance suppliers, study monitor, or regulatory authorities.
- Insufficient adherence to protocol requirements.

If the participation of the study site is terminated for reasons other than safety, the investigational drug substance suppliers will issue a written notice to the Investigator. The written notice will contain the reasons for taking such action. If the study site is terminated for non-compliance, appropriate regulatory authorities will also be notified by the Investigational drug substance suppliers.

# disclosure of data

## Confidentiality

Subject names will remain confidential and will not be included in the database supplied to the Investigational drug substance suppliers (or designee). Only subject number, subject initials, and birth date will be recorded on the CRF. If the subject name appears on any other document collected (e.g., hospital discharge summary), the name must be obliterated before the document is transmitted to the investigational drug substance suppliers (or designee). All study findings will be stored in electronic databases. The subjects will give explicit permission for representatives of the investigational drug substance suppliers, regulatory authorities, and the EC to inspect their medical records to verify the information collected. Subjects will be informed that all personal information made available for inspection will be handled in the strictest confidence and in accordance with appropriate laws and regulations. All personnel involved in the study will observe or work within the confines of the local data protection regulations.

The investigator will maintain a personal subject identification list (subject and treatment numbers with the corresponding subject names) to enable records to be identified.

## Publication

All information concerning the product as well as any information such as clinical indications for the drug, its formula, methods of manufacture and other scientific data relating to it, that have been provided by the investigational drug substance suppliers (or designee), and are unpublished, are confidential and must remain the sole property of the investigational drug substance suppliers. The PI will agree to use the information only for the purposes of carrying out this study and for no other purpose unless prior written permission from the investigational drug substance suppliers is obtained. The investigational drug substance suppliers have also full ownership of the CRFs completed as part of the study.

By signing the study protocol, the investigator agrees that the results of the study may be used for the purposes of national and international registration, publication, and information for medical and pharmaceutical professionals by the investigational drug substance suppliers. If necessary, the authorities will be notified of the investigator's name, address, qualifications, and extent of involvement.

The investigational drug substance suppliers (or designee) will prepare a final report on the study. The investigator may not publish or present any information on this study without the express written approval of the investigational drug substance suppliers. Additionally, the investigational drug substance suppliers, may, for any reason, withhold approval for publication or presentation for a period of three months.

# Investigator’s protocol agreement

The investigator's protocol agreement at the front of this document must be signed by the investigator/sponsor. An original or a copy must be kept on file at the investigational drug substance suppliers, and the investigator must retain an original or a copy. The completed protocol agreement signifies review and acceptance of the protocol by the investigator prior to initiation of the study.

# References

Arellano M, K Waller E. 2004. Granulocyte‑macrophage‑colony‑stimulating factor and other cytokines: as adjuncts to cancer immunotherapy, stem cell transplantation, and vaccines. Curr Hematol Rep. 3(6):424‑31.

Åsjö B, Stavang H, Sorensen B, Baksaas I, Nyhus J, Langeland N. 2002. Phase I trial of a therapeutic HIV type 1 vaccine, Vacc‑4x, in HIV type 1‑infected individuals with or without antiretroviral therapy. AIDS Res Hum Retroviruses. 18(18):1357‑65.

Banchereau J, Steinman RM. 1998. Dendritic cells and the control of immunity. Nature. 392(6673):245‑52.

Bernhardt SL, Gjertsen MK, Trachsel S, Moller M, Eriksen JA, Meo M, Buanes T, Gaudernack G. 2006. Telomerase peptide vaccination of patients with non‑resectable pancreatic cancer: A dose escalating phase I/II study. Br. J. Cancer. 95(11):1474‑82.

Brunsvig PF, Aamdal S, Gjertsen MK, Kvalheim G, Markowski‑Grimsrud CJ, Sve I, Dyrhaug M, Trachsel S, Moller M, Eriksen JA, Gaudernack G. 2006. Telomerase peptide vaccination: a Phase I/II study in patients with non‑small cell lung cancer. Cancer Immunol Immunother. 55(12):1553‑64.

Castro KG, Ward JW, Slutsker L, Buehler JW, Jaffe HW, Berkelman RL. 1992. 1993 Revised Classification System for HIV Infection and Expanded Surveillance Case Definition for AIDS Among Adolescents and Adults. MMWR. 41:1-19.

Dinoso JB et al. 2009. Treatment intensification does not reduce residual HIV-1 viremia in patients on highly active antiretroviral therapy. PNAS USA 106:9403–9408.

Disis ML, Bernhard H, Shiota FM, Hand SL, Gralow JR, Huseby ES, Gillis S, Cheever MA. 1996. Granulocyte‑macrophage colony-stimulating factor: an effective adjuvant for protein and peptide‑based vaccines. Blood. 88(1):202‑10.

Euroguidelines Group. 2007. European guidelines for the clinical management and treatment of HIV infected adults in Europe. European AIDS Clinical Society.

Forster SM, Osborne LM, Cheinsong‑Popov R, Kenny S, Burnell R, Jeffries DJ, [Jeffries DJ](http://www.ncbi.nlm.nih.gov/sites/?Db=pubmed&Cmd=Search&Term="Jeffries DJ"%5BAuthor%5D&itool=EntrezSystem2.PEntrez.Pubmed.Pubmed_ResultsPanel.Pubmed_RVAbstractPlus), [Pinching AJ](http://www.ncbi.nlm.nih.gov/sites/?Db=pubmed&Cmd=Search&Term="Pinching AJ"%5BAuthor%5D&itool=EntrezSystem2.PEntrez.Pubmed.Pubmed_ResultsPanel.Pubmed_RVAbstractPlus), [Harris JR](http://www.ncbi.nlm.nih.gov/sites/?Db=pubmed&Cmd=Search&Term="Harris JR"%5BAuthor%5D&itool=EntrezSystem2.PEntrez.Pubmed.Pubmed_ResultsPanel.Pubmed_RVAbstractPlus), [Weber JN](http://www.ncbi.nlm.nih.gov/sites/?Db=pubmed&Cmd=Search&Term="Weber JN"%5BAuthor%5D&itool=EntrezSystem2.PEntrez.Pubmed.Pubmed_ResultsPanel.Pubmed_RVAbstractPlus). 1987. Decline of anti‑p24 antibody precedes antigenaemia as correlate of prognosis in HIV‑1 infection. AIDS. 1(4):235‑40.

Gjertsen MK, Buanes T, Rosseland AR, Bakka A, Gladhaug I, Soreide O, Eriksen JA, Moller M, Baksaas I, Lothe RA, Seterdal I, Gaudernack G. 2001. Intradermal ras peptide vaccination with granulocyte‑macrophage colony‑stimulating factor as adjuvant: Clinical and immunological responses in patients with pancreatic adenocarcinoma. Int J Cancer. 92(3):441‑50.

Hodge JW, Greiner JW, Tsang KY, Sabzevari H, Kudo‑Saito C, Grosenbach DW, Gulley JL, Arlen PM, Marshall JL, Panicali D, Schlom J. 2006. Costimulatory molecules as adjuvants for immunotherapy. Front Biosci. 11:788‑803.

Jacobson JM, Lederman MM, Spritzler J, Valdez H, Tebas P, Skowron G, Wang R, Jackson JB, Fox L, Landay A, Gilbert MJ, O'Neil D, Bancroft L, Al-Harthi L, Jacobson MA, Merigan TC Jr, Glesby MJ. 2003. National Institute of Allergy and Infectious Diseases AIDS Clinical Trials Group. Granulocyte-macrophage colony-stimulating factor induces modest increases in plasma human immunodeficiency virus (HIV) type 1 RNA levels and CD4+ lymphocyte counts in patients with uncontrolled HIV infection. J Infect Dis. 188(12):1804‑14.

Kran AM, Sorensen B, Nyhus J, Sommerfelt MA, Baksaas I, Bruun JN, Kvale D. 2004. HLA‑ and dose‑dependent immunogenicity of a peptide‑based HIV‑1 immunotherapy candidate (Vacc‑4x). AIDS. 18(14):1875‑83.

Kvale D, Kran AM, Sommerfelt MA, Nyhus J, Baksaas I, Bruun JN, Sorensen B. 2005. Divergent in vitro and in vivo correlates of HIV‑specific T‑cell responses during onset of HIV viraemia. AIDS. 19(6):563‑67.

Panel of Antiretroviral Guidelines for Adults and Adolescents. 2007. Guidelines for the use of antiretroviral agents in HIV-1-infected adults and adolescents. December 1, 2007. Available at: <http://aidsinfo.nih.gov/>. Accessed 7 January 2008.

Robbins GK, Spritzler JG, Chan ES, Asmuth DM, Gandhi RT, Rodriguez BA, Skowron G, Skolnik PR,Shafer RW, Pollard RB, and the AIDS Clinical Trials Group 384 Team. 2009. Incomplete Reconstitution of T Cell Subsets on Combination Antiretroviral Therapy in the AIDS Clinical Trials Group Protocol 384. Clinical Infectious Disease. 48:350-61.

Sasaki MG, Foccacia R, de Messias-Reason IJ. 2003. Efficacy of granulocyte-macrophage colony-stimulating factor (GM-CSF) as a vaccine adjuvant for hepatitis B virus in patients with HIV infection. Vaccine. 21(31):4545-9.

Schneider E, Whitmore S, Glynn MK, Dominguez K, Mitsch A, McKenna MT. Revised surveillance case definitions for HIV Infection among adults, adolescents and children aged < 18 months and HIV infection and AIDS among children aged 18 months to < 13 years – United States, 2008. MMWR 2008; 57: RR-10.

Smart Study Group. 2006. The Strategies for Management of Antiretroviral Therapy (SMART) Study Group. CD4+ count‑guided interruption of antiretroviral treatment. N Engl J Med. 355:2283‑96.

Sommerfelt M, Nyhus J, Sørensen B. 2004. Novel peptide-based HIV-1 immunotherapy. Expert Opinion on Biological Therapy. 4 (3):349-61.

Trono D et al. 2010. HIV persistence and the prospect of long-term drug-free remissions for HIV-infected individuals. Science 329: 174-180.

Visionar Final Report of Study VP07-07 (2007). Repeated dose toxicity in rats after nasal administration of an adjuvanted nasal influenza vaccine of serotype H1N1.

Visionar Final Report of Study VP-08-35-01 (2009). Repeated dose toxicity in rats after nasal administration of an influenza vaccine of serotype H1N1 with anionic adjuvant as adjuvant.

Weber JN, Clapham PR, Weiss RA, Parker D, Roberts C, Duncan J, Weller I, Carne C, Tedder RS, Pinching T, Chiengsong-Popov R. 1987. Human immunodeficiency virus infection in two cohorts of homosexual men: neutralising sera and association of anti‑gag antibody with prognosis. Lancet. 1:119‑22.

# appendices

## Division of AIDS Table for Grading Severity of Adult and Pediatric Adverse Events, December 2004

## AIDS-Defining Illnesses

- Candidiasis of bronchi, trachea, or lungs
- Candidiasis, esophageal
- Cervical cancer, invasive
- Coccidioidomycosis, disseminated, or extra pulmonary
- Cryptococcosis, extra pulmonary
- Cryptosporidiosis, chronic intestinal (>1 month’s duration)
- Cytomegalovirus disease (other than liver, spleen or nodes)
- Cytomegalovirus retinitis (with loss of vision)
- Encephalopathy, HIV-related
- Herpes simplex: chronic ulcer(s) (>1 month’s duration); or bronchitis, pneumonitis, or esophagitis
- Histoplasmosis, disseminated, or extra pulmonary
- Isosporiasis, chronic intestinal (>1 month’s duration)
- Kaposi’s sarcoma
- Lymphoma, Burkitt’s (or equivalent term)
- Lymphoma, immunoblastic (or equivalent term)
- Lymphoma, primary, of brain
- *Mycobacterium avium* complex or M. kansasii, disseminated, or extra pulmonary
- Mycobacterium tuberculosis, any site (pulmonary or extra pulmonary)
- Mycobacterium, other species or unidentified species disseminated or extra pulmonary
- *Pneumocystis carinii* pneumonia
- Pneumonia, recurrent
- Progressive multifocal leukoencephalopathy
- Salmonella septicemia, recurrent
- Toxoplasmosis of brain
- Wasting syndrome due to HIV

For more information refer to Castro, 1993 and Schneider, 2008.
